# Supplementary material for: Fitness, strength and severity of COVID-19: a prospective register study of 1 559 187 Swedish conscripts
Source: BMJ Open. 2021 Jul 5;11(7):e051316. doi: 10.1136/bmjopen-2021-051316 (PMC8260308; doi:10.1136/bmjopen-2021-051316)
Supplement: Supplementary data [file bmjopen-2021-051316supp001.pdf]

Supplementary material

Table S1. Included main diagnoses divided by categories when COVID-19 is secondary diagnosis.

Included categories	ICD-codes
**COVID-related symptoms**	
Cough	R05
Abnormalities of breathing	R06
Pain in throat and chest	R07
Other symptoms and signs involving the circulatory and respiratory system	R09
Dizziness and giddiness	R42
Fever of other or unknown origin	R50
Headache	R51
Malaise and fatigue	R53
Syncope and collapse	R55
**Upper and lower respiratory tract infections**	
Acute nasopharyngitis	J00
Acute tonsillitis	J03
Acute upper respiratory infections of multiple and unspecified sites	J06
Influenza due to other identified influenza virus	J10
Other viral pneumonia	J128
Viral pneumonia, unspecified	J129
Pneumonia due to Streptococcus pneumoniae	J13
Bacterial pneumonia, not elsewhere classified	J15
Pneumonia due to other specified infectious organisms	J168
Pneumonia in diseases classified elsewhere	J17
Pneumonia, unspecified organism	J18
Unspecified acute lower respiratory infection	J22
Coronavirus infection, unspecified	B342
Other viral infections of unspecified site	B348
Viral infection, unspecified	B349
Coronavirus as the cause of diseases classified elsewhere	B972
Other and unspecified infectious diseases	B99
**Respiratory disorders**	
Pulmonary embolism	I26
Acute respiratory distress syndrome	J80
Pulmonary edema	J81
Pleural effusion not elsewhere classified	J90
Respiratory failure, not elsewhere classified	J96
Respiratory disorders in diseases classified elsewhere	J99
**Obstructive Airway Diseases**	
Acute bronchitis due to other specified organisms	J208
Acute bronchitis, unspecified	J209
Acute bronchiolitis due to other specified organisms	J218
Acute bronchiolitis, unspecified	J219
Other chronic obstructive pulmonary disease	J44
Asthma	J45
**Cardiac diseases**	
Viral carditis	B332
Chronic ischemic heart disease	I25
Acute pericarditis	I30
Pericarditis in diseases classified elsewhere	I32
Acute myocarditis, unspecified	I40
Myocarditis in diseases classified elsewhere	I41
Atrial fibrillation and flutter	I48

Heart failure	I50
Abnormalities of heart beat	R00
**Kidney disorders**	
Acute kidney failure	N17
Chronic kidney disease	N18
Unspecified kidney failure	N19
**Electrolyte disorders**	
Other disorders of fluid, electrolyte and acid-base balance	E87

Table S2: Results of main analysis. Association between conscript's fitness at time of examination and hospitalization, intensive care and death due to COVID-19 (OR, 95% CI, Firth's bias correction).

Fitness level	**Basic model[a] n=1 143 670**	**Basic model controlled for BMI[b] n=1 143 670**	**Basic model controlled for blood pressure n=1 140 514**	**Basic model controlled for baseline morbidity[c] n=1 143 670**	**Basic model controlled for BMI^2 and height N=1 143 670**	**Basic model controlled for parental education n=1 031 246**	**Basic model controlled for BMI, height, blood pressure, morbidity and parental education n=1 028 122**
	**All hospitalization due to COVID-19.**						
	cases=2 006	cases=2 006	cases=2 003	cases=2 006	cases=2 006	cases=1 710	cases=1 707
Low (1-5)	**1**	**1**	**1**	**1**	**1**	**1**	**1**
Medium (6-7)	0.91 (0.82-1.01)	0.92 (0.83-1.01)	0.91 (0.82-1.01)	0.91 (0.82-1.01)	0.91 (0.82-1.01)	0.91 (0.82-1.02)	0.91 (0.81-1.02)
High (8-9)	**0.76** (0.67-0.85)	**0.73** (0.65-0.83)	**0.76** (0.68-0.85)	**0.76** (0.67-0.85)	**0.71** (0.63-0.81)	**0.74** (0.65-0.85)	**0.73** (0.64-0.83)
AUROC	0.66	0.66	0.66	0.66	0.66	0.67	0.67
	**Intensive care due to COVID-19**						
	cases=445	cases=445	cases=445	cases=445	cases=445	cases=363	cases=363
Low (1-5)	**1**	**1**	**1**	**1**	**1**	**1**	**1**
Medium (6-7)	**0.76** (0.62-0.95)	**0.77** (0.62-0.95)	**0.76** (0.62-0.95)	**0.77** (0.62-0.95)	**0.76** (0.61-0.94)	**0.76** (0.60-0.96)	**0.76** (0.60-0.96)
High (8-9)	**0.61** (0.48-0.78)	**0.58** (0.45-0.75)	**0.61** (0.48-0.78)	**0.62** (0.48-0.79)	**0.57** (0.44-0.74)	**0.60** (0.45-0.80)	**0.60** (0.46-0.80)
AUROC	0.69	0.71	0.70	0.69	0.71	0.71	0.71
	**Mortality due to COVID-19**						
	cases=149	cases=149	cases=149	cases=149	cases=149	cases= 111	cases=111
Low (1-5)	**1**	**1**	**1**	**1**	**1**	**1**	**1**
Medium (6-7)	**0.67** (0.46-0.96)	**0.64** (0.44-0.94)	**0.67** (0.47-0.97)	**0.66** (0.46-0.96)	**0.63** (0.43-0.92)	**0.53** (0.34-0.83)	**0.53** (0.34-0.81)
High (8-9)	**0.56** (0.37-0.85)	**0.52** (0.34-0.80)	**0.57** (0.38-0.87)	**0.56** (0.37-0.85)	**0.50** (0.32-0.77)	**0.52** (0.32-0.83)	**0.50** (0.31-0.81)
AUROC	0.81	0.82	0.81	0.81	0.82	0.82	0.82

[a] adjusted for year and place of examination, and age at examination.
[b] BMI, BMI^2, BMI^3
[c] including CVD, diabetes, kidney disease, respiratory disease and cancer.

Table S3: Association between muscle strength at conscription and severity of COVID disease (OR for disease by 1 SD higher muscle strength (SD = 313 N), 95% CI, Firth's bias correction)

Outcome	Total n	Cases	OR (95% CI)	AUROC
Hospital[a]	1 161 914	2 252	**0.89** (0.84-0.93)	0.64
ICU[a]		514	**0.88** (0.79-0.97)	0.68
Mortality[a]		180	**0.83** (0.70-0.99)	0.80
Hospital[b]	952 225	1 850	**0.90** (0.86-0.95)	0.64
ICU[b]		421	0.90 (0.80-1.01)	0.68
Mortality[b]		146	0.85 (0.70-1.03)	0.79

[a] basic model adjusted for BMI, BMI^2, BMI^3 and height.
[b] above model further adjusted for fitness categories.

Figure S1: Results of restricted cubic spline analyses of continuous muscle strength and severity of COVID-19 adjusted for age, year, and place of conscript examination, BMI, BMI², BMI³, and height (total number of observations = 1 161 914).

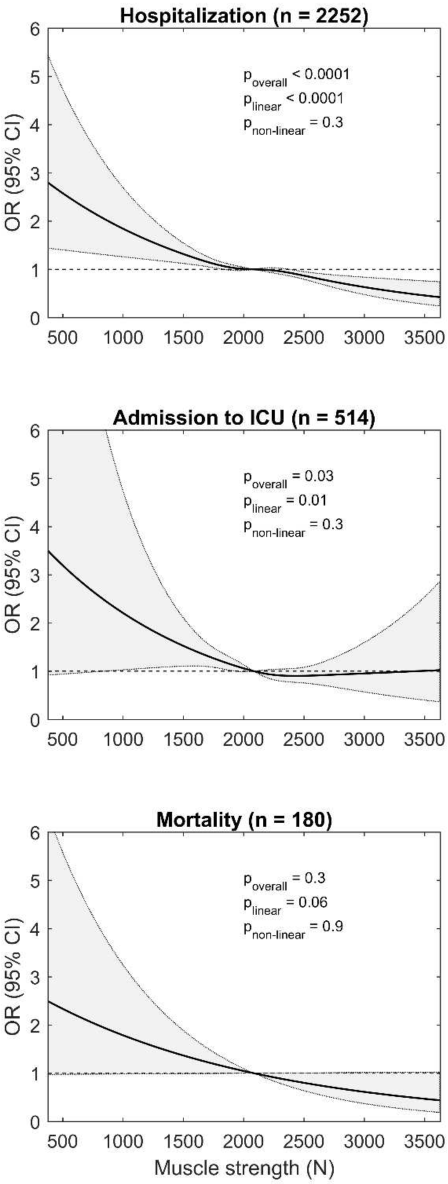

Table S4. Results of mediation analysis between cardiovascular fitness, muscle strength, cardiovascular disease and severe COVID-19 (OR with 95%CI).

Outcome	Direct	Indirect	Total	Proportion-mediated[c]
	**Muscle strength[a]**			
Hospital	**0.893** (0.850-0.937)	**0.995** (0.994-0.996)	**0.888** (0.846-0.933)	4.0 %
ICU	**0.880** (0.796-0.973)	**0.997** (0.995-0.998)	**0.877** (0.793-0.970)	2.2 %
Mortality	0.855 (0.720-1.015)	**0.992** (0.989-0.995)	0.848 (0.714-1.007)	4.5 %
	**CRF[a,b]**			
Hospital[a]	**0.860** (0.810-0.913)	**0.987** (0.985-0.989)	**0.849** (0.800-0.901)	7.4 %
ICU[a]	**0.772** (0.679-0.877)	**0.991** (0.987-0.995)	**0.765** (0.673-0.869)	3.0 %
Mortality[a]	**0.725** (0.578-0.909)	**0.978** (0.969-0.986)	**0.709** (0.566-0.889)	5.5 %

[a] adjusted for age, year, and place of conscript examination, BMI, BMI^2, BMI^3 and height.
[b] treating the 3-level variable for CRF as ordinal score.
[c] calculated from direct and indirect effect odds ratios as $OR^{direct}(OR^{indirect} - 1)/(OR^{direct}OR^{indirect} - 1)$.

VanderWeele T, Mediation Analysis: A Practioner's Guide, Annu. Rev. Public Health 2016.37:17-32
