# Supplementary material for: Fitness, strength and severity of COVID-19: a prospective register study of 1 559 187 Swedish conscripts
Source: BMJ Open. 2021 Jul 5;11(7):e051316. doi: 10.1136/bmjopen-2021-051316 (PMC8260308; doi:10.1136/bmjopen-2021-051316)
Supplement: Author's manuscript [file bmjopen-2021-051316.draft_revisions.pdf]

BMJ Open is committed to open peer review. As part of this commitment we make the peer review history of every article we publish publicly available.

When an article is published we post the peer reviewers' comments and the authors' responses online. We also post the versions of the paper that were used during peer review. These are the versions that the peer review comments apply to.

The versions of the paper that follow are the versions that were submitted during the peer review process. They are not the versions of record or the final published versions. They should not be cited or distributed as the published version of this manuscript.

BMJ Open is an open access journal and the full, final, typeset and author-corrected version of record of the manuscript is available on our site with no access controls, subscription charges or pay-per-view fees (http://bmjopen.bmj.com).

If you have any questions on BMJ Open's open peer review process please email info.bmjopen@bmj.com

BMJ Open

# Fitness and strength in young Swedish men predicted incidence of severe COVID-19 during the first wave of the pandemic – a prospective register study

| | |
|---|---|
| Journal: | *BMJ Open* |
| Manuscript ID | bmjopen-2021-051316 |
| Article Type: | Original research |
| Date Submitted by the Author: | 15-Mar-2021 |
| Complete List of Authors: | af Geijerstam, Agnes; University of Gothenburg Sahlgrenska Academy, School of public health and community medicine, department of medicine<br>Mehlig, Kirsten; University of Gothenburg Sahlgrenska Academy, School of Public Health and Community Medicine<br>Börjesson, Mats; University of Gothenburg Sahlgrenska Academy, Dept of Molecular and Clinical Medicine; University of Gothenburg, Center for Health and Performance<br>Robertson, Josefina; University of Gothenburg Sahlgrenska Academy, School of Public Health and Community Medicine; University of Gothenburg Sahlgrenska Academy, Department of Infectious Diseases, Institute of Biomedicine<br>Nyberg, Jenny; University of Gothenburg Sahlgrenska Academy, Section for Clinical Neuroscience, Institute of Neuroscience and Physiology<br>Adiels, Martin; University of Gothenburg Sahlgrenska Academy, School of Public health and Community Medicine; University of Gothenburg Sahlgrenska Academy, Dept of Molecular and Clinical Medicine<br>Rosengren, Annika ; University of Gothenburg Sahlgrenska Academy, Department of Molecular and Clinical Medicine; Sahlgrenska University Hospital<br>Åberg, Maria; University of Gothenburg Sahlgrenska Academy, a School of Public Health and Community Medicine, Institute of Medicine; Region Västra Götaland, Regionhälsan<br>Lissner, Lauren; University of Gothenburg Sahlgrenska Academy, School of Public Health and Community Medicine, Institute of Medicine |
| Keywords: | COVID-19, EPIDEMIOLOGY, Public health < INFECTIOUS DISEASES |
| | |

SCHOLARONE™
Manuscripts

*I, the Submitting Author has the right to grant and does grant on behalf of all authors of the Work (as defined in the below author licence), an exclusive licence and/or a non-exclusive licence for contributions from authors who are: i) UK Crown employees; ii) where BMJ has agreed a CC-BY licence shall apply, and/or iii) in accordance with the terms applicable for US Federal Government officers or employees acting as part of their official duties; on a worldwide, perpetual, irrevocable, royalty-free basis to BMJ Publishing Group Ltd ("BMJ") its licensees and where the relevant Journal is co-owned by BMJ to the co-owners of the Journal, to publish the Work in this journal and any other BMJ products and to exploit all rights, as set out in our licence.*

*The Submitting Author accepts and understands that any supply made under these terms is made by BMJ to the Submitting Author unless you are acting as an employee on behalf of your employer or a postgraduate student of an affiliated institution which is paying any applicable article publishing charge ("APC") for Open Access articles. Where the Submitting Author wishes to make the Work available on an Open Access basis (and intends to pay the relevant APC), the terms of reuse of such Open Access shall be governed by a Creative Commons licence – details of these licences and which Creative Commons licence will apply to this Work are set out in our licence referred to above.*

*Other than as permitted in any relevant BMJ Author's Self Archiving Policies, I confirm this Work has not been accepted for publication elsewhere, is not being considered for publication elsewhere and does not duplicate material already published. I confirm all authors consent to publication of this Work and authorise the granting of this licence.*

For peer review only

**SCHOOL OF PUBLIC HEALTH AND COMMUNITY MEDICINE**

Dear Editor,

Please consider for publication our manuscript *Fitness and strength in young Swedish men predicted incidence of severe COVID-19 during the first wave of the pandemic – a prospective register study.*

Here we show that there is a strong protective association between higher physical fitness during late adolescence and early adulthood and later severity of COVID-19, using the Swedish Military conscripts registry and national health registries.

Physical fitness has been shown to affect a large number of health outcomes, and the results of this study reinforces this notion as it highlights the importance of fitness in youth.

There is to our knowledge no other comparable study.

The manuscript represents original work and has not been published previously. The manuscript is currently not being considered for publication by another journal. Each author has made substantial contributions and approved of the submitted manuscript.

Agnes af Geijerstam, M.D., doctoral student

School of Public Health and Community Medicine, Institute of Medicine, Sahlgrenska Academy, University of Gothenburg, Gothenburg, Sweden

School of Public Health and Community Medicine, Institute of Medicine, University of Gothenburg
Medicinaregatan 18A, PO Box 463, SE 405 30 Gothenburg, Sweden
+46 31 786 00 00
www.gu.se



For peer review only - http://bmjopen.bmj.com/site/about/guidelines.xhtml

**Fitness and strength in young Swedish men predicted incidence of severe COVID-19 during the first wave of the pandemic – a prospective register study.**

Authors: Agnes af Geijerstam [a], Kirsten Mehlig [a], Mats Börjesson [b, c ,d], Josefina Robertson [a, d, e], Jenny Nyberg [f], Martin Adiels [a, b] , Annika Rosengren [b, d], Maria Åberg [a, g], Lauren Lissner [a]

[a] School of Public Health and Community Medicine, Institute of Medicine, Sahlgrenska Academy, University of Gothenburg, Gothenburg, Sweden

[b] Dept of Molecular and Clinical Medicine, Sahlgrenska Academy; University of Gothenburg

[c] Center for Health and Performance, University of Gothenburg

[d] Sahlgrenska University Hospital, Region Västra Götaland, Gothenburg, Sweden.

[e] Department of Infectious Diseases, Institute of Biomedicine, Sahlgrenska Academy, University of Gothenburg, Gothenburg, Sweden

[f] Section for Clinical Neuroscience, Institute of Neuroscience and Physiology, Sahlgrenska Academy, University of Gothenburg, Gothenburg, Sweden

[g] Region Västra Götaland, Regionhälsan, Gothenburg, Sweden

**Corresponding author:** Agnes af Geijerstam, School of Public Health and Community Medicine, Institute of Medicine, Sahlgrenska Academy, University of Gothenburg, P.O Box 459, 405 30 Gothenburg, Sweden. agnes.af.geijerstam@gu.se

**Acknowledgements:** This work was supported by grants from supported by grants from the Swedish ALF-agreement (ALFGBG-720201) and the Swedish Research Council (02508, VRREG 2019-00193, 2020-05792). The funding sources had no role in study design, collection, analysis and interpretation of data, the writing of the report, or the decision to submit the article for publication.

**Competing interest statement**: All authors have completed the Unified Competing Interest form (available on request from the corresponding author) and declare: no support from any organization for the submitted work; no financial relationships with any organizations that might have an interest in the submitted work in the previous three years, no other relationships or activities that could appear to have influenced the submitted work.

**Transparency statement: AG** affirms that the manuscript is an honest, accurate, and transparent account of the study being reported and that no important aspects of the study have been omitted.

**Dissemination to participants and related patient and public communities:** We will make the findings of this article public with a press release and via media contacts.

**Keywords:** COVID-19, physical fitness, cardiorespiratory fitness, muscular strength, adolescence, men.



For peer review only - http://bmjopen.bmj.com/site/about/guidelines.xhtml

**Abstract**

**Objective** Investigating the possible connection between cardiorespiratory fitness and muscle strength in early adulthood and severity of COVID-19 later in life.

**Design** Prospective registry based cohort study.

**Participants** 1 559 187 Swedish men, undergoing military conscription between 1968 and 2005 at a mean age of 18.3 (SD 0.73) years.

**Main outcome measures** Hospitalization, intensive care or death due to COVID-19 from March to September 2020, in relation to three categories of cardiorespiratory fitness (CRF) and muscle strength

**Results** High cardiorespiratory fitness in late adolescence and early adulthood was protective against severe COVID-19 later in life with OR (95% CI) 0.75 (0.66-0.85) for hospitalization (n=2 594), 0.62 (0.48-0.82) for intensive care (n=559), and 0.56 (0.37-0.85) for mortality (n=184), compared to the lowest category of CRF. The effect remains unchanged when controlled for body mass index (BMI), blood pressure, parental education level and incident cardiovascular disease before 2020. Moreover lower muscle strength in late adolescence showed a linear association with a higher risk of all three outcomes when controlled for BMI.

**Conclusions** Physical fitness at a young age is associated with severity of COVID-19 many years later. This underlines the necessity to increase the general physical fitness of the population to offer protection against coming viral pandemics.

STRENGHTS AND LIMITATIONS OF THIS STUDY

# Using data from the Swedish military conscript registry provided us with objective measures of fitness in a uniquely large sample.

# The prospective design with long and near-complete follow-up from well validated hospital records and the death registry provides a strong ground for drawing conclusions.

# Limitations include the fact that the results from this cohort can only be generalized to men, since the conscription process was not mandatory for women.

# Also during the last decade the lowest cardiorespiratory fitness scores were not recorded in the registry, making it difficult to draw conclusions regarding very low CRF, and maybe underestimating the adverse association.



For peer review only - http://bmjopen.bmj.com/site/about/guidelines.xhtml

## Background

The COVID-19 pandemic has affected the world in unprecedented ways. While measures such as contact restrictions and vaccinations relieve the immediate impact of the disease, epidemiologic studies may identify risk factors that could be managed in the long run, in order to reduce the impact of future epidemics. Early on, advanced age and current cardiovascular comorbidities including obesity were found to be associated with a more severe course of disease(1).

Cardiorespiratory fitness is regarded as one of the most important risk factors for overall health, cardiovascular morbidity and overall mortality(2). The level of physical activity early in life (and associated cardiorespiratory fitness) may infer short and long-term effects on risk factors and the immune system. There has been speculation about the way it may confer protection against severe COVID-19(3). An early observation from the UK Biobank cohort that slower self-reported walking pace predicted more severe COVID-19 suggests the importance of cardiorespiratory fitness for this particular disease(4). Although cardiorespiratory fitness is a persistent trait(3) with a genetic component(5), it is also to a large extent modifiable by regular exercise. Overall, physical fitness consists of cardiorespiratory fitness and muscular fitness, including muscle strength and healthy weight. BMI is routinely measured in hospital and public care settings and has already been shown to be associated with severity of disease(1). However, objective measures of cardiorespiratory fitness and strength before the onset of COVID-19 is rarely available in population based studies. Self-reported measures are unreliable and most studies conducted in middle-age or older populations have limited information on risk factors in earlier life(6). Participation rates among young men in population-based studies also tend to be low(7).

The Swedish Military Conscripts registry contains detailed and high-quality information about cardiorespiratory fitness and strength in a uniquely large sample of young men. In contrast to many population-based surveys, the participation rates until the early 2000's yielded highly generalizable results. Combined with measured data on BMI and other potential confounding factors in early adulthood, linkage data from nation-wide disease registries enables a life-course perspective on the role of physical fitness as a potentially mitigating factor for severe COVID-19 that is presently lacking in the literature.

## Methods

### Study design

This prospective cohort study is based on data from the Swedish military conscript registry, the longitudinal integration database for health insurance and labor market studies (LISA registry), and the Swedish national hospital, intensive care and cause of death registries (for description in detail, see Åberg et al, 2015). Fitness data was collected in early adulthood, in relation to incidence data on COVID-19 retrieved in September 2020, after the first wave of the pandemic in Sweden.

As this is a registry based study, there have been no patient or public involvement.



For peer review only - http://bmjopen.bmj.com/site/about/guidelines.xhtml

*Population studied*

The Swedish military conscript's registry contains information about 1 938 027 Swedish individuals who enlisted for military service between late 1968 and 2005 giving between 15-52 years of follow-up until the outbreak of COVID-19. During that time, Swedish law required all male citizens to enlist, except for those in prison or those with severe chronic somatic or psychiatric conditions or functional disabilities (approximately 2-3% annually). Due to a shift to voluntary recruitment in 2005 the data in later years is no longer considered representative. The standardized protocol included measurements of weight and height, blood pressure, muscular strength and cardiorespiratory fitness. The examinations took place over two days in six conscription centers across Sweden.

**Main independent variables**

*Cardiorespiratory fitness score*

To evaluate cardiorespiratory fitness (CRF) the subjects performed a cycle ergometric test where the work rate was successively increased until limited by exhaustion. Based on the final work rate, a 9-level score derived by the National Service Administration in Sweden(8) served as a standardized measure of CRF. Due to changes in the conscription examination protocols, raw data were not available for all conscripts(9). However, CRF scores matching certain military requirements were assigned during all recruitment years, and can be assumed to be comparable across years(5). For this study, the scores were collapsed into three categories describing low (1-5), medium (6-7) and high (8-9) CRF. The asymmetrical assignment of CRF scores was prompted by the fact that the fitness data in the lowest categories were omitted from the registry after 1999(10).

*Muscle strength score*

Isometric muscle strength in units of Newton was assessed as a weighted sum of knee extension (weight 1.3), elbow flexion (weight 0.8) and handgrip (weight 1.7). The underlying value for isometric muscle strength was available in the registry until 1996, making it possible to examine strength as a continuous variable(8).

**Other covariates**

*BMI and weight status*

Weight and height were measured by standard anthropometric measurement techniques, continuous BMI values ($kg/m^2$) were calculated, and also divided into categories of underweight (BMI <18.5), normal weight (BMI 18.5-24.9), overweight (BMI 25.0-29.9), obesity (BMI 30-34.9) and obesity class 2 (BMI ≥35)(11). For those aged 15 to 17 at conscription weight status categories were adjusted based on age- and sex-specific BMI z-scores, according to the International childhood BMI cutoffs (IOTF)(12).

*Blood pressure*



For peer review only - http://bmjopen.bmj.com/site/about/guidelines.xhtml

High blood pressure in early adulthood was used as an indicator of predisposition to later hypertension and other cardiovascular disease(6). Systolic and diastolic blood pressure was measured according to a standardized protocol after five to 10 minutes of rest in the supine position. In this study, values were then categorized into five subgroups (optimal blood pressure (systolic blood pressure <120 mm Hg and diastolic blood pressure <80 mm Hg), normal blood pressure (120-129 and 80-84 mm Hg), high normal blood pressure (130-139 and 85-89 mm Hg), grade 1 hypertension (140-159 and 90-99 mm Hg), and grade 2 hypertension (≥160 and ≥100 mm Hg) based on the 2018 European guidelines(13).

*Parental education*

Parental education was considered a proxy for socioeconomic position of the family of origin. Using data from the LISA registry, parental education was based on the highest of maternal and paternal education, and divided into three categories with low education (up to 9 years), medium education (high school diploma with ≤2 years at university) and high (≥3 years at university)(9).

**Analytic samples and subsamples**

Figure 1 describes the construction of the analytic sample. Among 1 949 891 individuals examined for conscription between 1968 and 2005, we excluded all females, those with ambiguous identification numbers, without known place or year of conscription, and death or emigration without return before 2020, leaving 1 737 217 observations. Because of the aim of relating fitness measured in adolescence and early adulthood to disease severity later in life, individuals with age at conscription outside the interval of 15 to 30 were excluded. Since BMI was an important confounder for the association between fitness and disease, the sample was further restricted excluding all men with missing or implausible BMI-values (range for inclusion set between 15 and 60). Among the remaining 1 559 187 men, 1 143 670 had values for CRF and 1 161 914 had registered values for the continuous measure of muscle strength.

[Figure 1 about here]

**Outcome parameters: Hospitalization and/or death due to COVID-19**

Using the Swedish personal identification number the full sample was linked to the national hospital, the intensive care and the cause of death registries. From these, all cases between March and September 2020 with a main diagnosis of ICD U071 for test verified infection with SARS-CoV-2 and U072 for clinically diagnosed COVID-19, or deaths with U071 or U072 as an underlying cause of death were identified.

Records with U071 or U072 as a secondary diagnosis were counted as cases if the main diagnosis was clinically related to COVID-19 (full list of diagnoses in supplementary materials). This resulted in a reduction of total cases from 2 957 to 2 635.

All cases were considered as severe COVID-19 since they were requiring hospital care. Furthermore, patients were categorized into three groups with increasing severity;



For peer review only - http://bmjopen.bmj.com/site/about/guidelines.xhtml

For peer review only

(I) Hospitalization due to COVID-19, n = 2594

(II) Intensive care due to COVID-19, n = 559

(III) Deaths due to COVID-19, n = 184

**Statistical analysis**

The main independent variable, cardiorespiratory fitness, was analyzed in terms of three categories describing low, medium, and high values of exposure. Logistic regression was used to calculate the odds for COVID-19 outcome by exposure category, adjusted for regional examination center, age at and year of conscription, as well as linear, quadratic and cubic terms of BMI, parental education and blood pressure category at examination. Effect modification was investigated by including product terms between exposure categories (CRF categories) and categorical predictors into the model, overweight status (BMI ≥ 25 kg/m$^2$), parental education, blood pressure category, and current age dichotomized at median value (52 years). For each interaction analysis, the p-value from an F-test comparing a model with and without interactions was assessed. Results were given in terms of odds ratios (OR) and 95% confidence intervals (95% CI). The area under the receiver operating characteristics curve (AUROC) was used as a measure for the discriminative properties of the logistic models. Muscle strength in units of Newton (N) was standardized to zero mean and unit standard deviation (SD), and results were given in terms of OR per SD. To explore the non-linear association between muscle strength and Covid19 we used logistic regression based on restricted cubic splines(14). Statistical analyses were performed with SAS version 9.4 (SAS Institute, Cary, NC). Statistical significance was set at 0.05 (2-sided tests).

*Sensitivity analysis*

To investigate whether the effects of high CRF or strength was explained by less cardiovascular disease (CVD) during midlife, we excluded all cohort members with an intermediate CVD diagnosis (I00-I99 (ICD 10) and 390-459 (ICD7-9) up to December 2019).

**Ethics statement**

The study conforms to the principles outlined in the Declaration of Helsinki. The Ethics Committee of the University of Gothenburg and Confidentiality Clearance at Statistics Sweden approved the study (EPN Reference numbers EPN 462-14 and 567-15; T174-15, T653-17, T196-17, T 2020-01325, T 2020-02420.). The analysis included here is based on data from 15-30 year olds in the study population defined by that application, i.e. examined between 1968 and 2005. The requirement for informed consent was waived for secondary analysis of existing data.

**Results**

Basic characteristics of the 1 559 187 men included in the study are shown by conscription decade in table 1. The majority of deaths and hospitalizations due to COVID-19 were observed among those who underwent conscription between 1968 and 1985.



For peer review only - http://bmjopen.bmj.com/site/about/guidelines.xhtml

**Table 1**: Description of analytical sample, by conscription decade.

| **Conscription year** | 1968-1975 | 1976-1985 | 1986-1995 | 1996-2005 | All decades |
|---|---|---|---|---|---|
| N total | 283 082 | 441 414 | 496 484 | 338 207 | 1 559 187 |
| **Age** | | | | | |
| at conscription, mean (SD) | 18.5 (0.65) | 18.3 (0.82) | 18.3 (0.76) | 18.2 (0.58) | 18.3 (0.73) |
| in 2020 (mean, SD) | 65.9 (1.94) | 57.5 (2.98) | 48.0 (3.00) | 38.3 (2.91) | 51.8 (9.89) |
| **CRF** | | | | | |
| Total | 248 424 | 380 791 | 358 731 | 155 720 | 1 143 670 |
| CRF low n (%) | 89 953 (36.2) | 135 569 (35.6) | 85 203 (23.8) | 25 546 (16.4) | 336 271 (29.4) |
| CRF medium n (%) | 84 189 (33.9) | 127 965 (33.6) | 189 000 (52.7) | 87 343 (56.1) | 488 497 (42.7) |
| CRF high n (%) | 74 286 (29.9) | 117 257 (30.8) | 84 528 (23.6) | 42 831 (27.5) | 318 902 (27.9) |
| **Muscle strength (N)** | | | | | |
| Total | 277 150 | 435 32 | 449 188 | n/a | 1 161 661 |
| Mean (SD) | 2 033 (299) | 2 092 (315) | 2 140 (315) | n/a | 2 096 (313) |
| **BMI** | | | | | |
| Mean (SD) | 21.2 (2.6) | 21.7 (2.8) | 22.0 (3.0) | 22.7 (3.6) | 21.9 (3.0) |
| Overweight and obese n (%) | 20 481 (1.5) | 44 265 (2.2) | 65 268 (3.2) | 64 943 (5.6) | 194 957 (3.6) |
| **Parental education** | | | | | |
| Low n (%) | 101 918 (52.9) | 141 205 (34.8) | 85 586 (17.5) | 28 186 (8.4) | 356 895 (25) |
| Medium n (%) | 77 020 (40) | 213 050 (52.5) | 309 246 (63.1) | 222 095 (65.8) | 821 411 (57.6) |
| High n (%) | 13 787 (7.2) | 51 660 (12.7) | 95 589 (19.5) | 87 069 (25.8) | 248 105 (17.4) |
| **Blood pressure** | | | | | |
| Grade 1 hypertension, n (%) | 51 242 (18.1) | 81 990 (18.6) | 89 752 (18.5) | 57 582 (21.4) | 280 566 (19) |
| Grade 2 hypertension, n (%) | 2 167 (0.8) | 3 145 (0.7) | 2 685 (0.6) | 1 614 (0.6) | 9 611 (0.7) |
| **COVID-19 diagnoses** | | | | | |
| n in hospital due to COVID-19 (%) | 728 (0.26) | 944 (0.21) | 674 (0.14) | 248 (0.07) | 2 594 |
| n in intensive care due to COVID-19 (%) | 195 (0.07) | 209 (0.05) | 130 (0.03) | 25 (0.01) | 559 |
| n dead due to COVID-19 (%) | 117 (0.04) | 43 (0.01) | 24 (0.00) | 0 | 184 |



For peer review only - http://bmjopen.bmj.com/site/about/guidelines.xhtml

*Cardiorespiratory fitness in early adulthood and severity of COVID-19*

Our analysis shows a protective association between higher cardiorespiratory fitness at conscription and hospitalization, intensive care or death due to COVID-19 later in life, adjusted for examination year and place and conscript's age at examination. OR for high versus low fitness were 0.75 (95% CI 0.66-0.85), 0.62 (0.48-0.82) and 0.56 (0.37-0.85) respectively.

This association remains statistically significant with only minor attenuation after also controlling for BMI (figure 2, left panel), blood pressure and parental education level (full results in table S1, supplementary materials).

[figure 2 about here]

In order to examine whether there was any further protection at the highest level of CRF, categories 8 and 9 were disaggregated but no statistically significant difference could be seen between the two subgroups. Adjusting for muscle strength did not change the results for CRF.

The associations between CRF and all three COVID-19 endpoints were not modified by weight status, blood pressure or education. For example, comparing high vs. low fitness in overweight (BMI group 3-5) and non-overweight conscripts (BMI group 1-2), the association with intensive care requiring COVID-19 was stronger in the overweight group, OR = 0.49 (0.25-0.96), than in the non-overweight group, OR = 0.65 (0.50-0.85), but not significantly so (p-value for interaction = 0.5).

*Muscle strength and severity of COVID-19*

Muscular strength, a continuous variable measured in units of Newton and recorded until 1997, showed associations with Covid-19 that were similar in direction to the more robust protective effects of cardiorespiratory fitness. As shown in figure 2, right panel, when analyzed in intervals of 313 N (=1 SD) the association was statistically significant for hospitalization and intensive care, but after controlling for cardiovascular fitness the results were attenuated (full results in table S2). Further analysis using cubic spline analyses confirmed the association between muscle strength and Covid-19. Specifically, these analyses, illustrated in supplementary materials (figure S1), confirmed that lower muscle strength in young adulthood has a significant linear association for all 3 endpoints, and no significant non-linear component.

*Results of sensitivity analysis*

Exclusion of all COVID-19 cases and controls with incident CVD between conscription and 2019 reduced the sample of around one third of the hospitalized patients and half of the ones who died. However, even in the remaining analytic sample the effect sizes of high CRF remains significant, although



For peer review only - http://bmjopen.bmj.com/site/about/guidelines.xhtml

with some attenuation. OR for high versus low fitness were 0.74 (0.64-0.86) (hospitalization), 0.63 (0.45-0.86) (intensive care) and 0.33 (0.16-0.72) (death). For muscular strength the association remains as well but is weakened when adjusted for CRF in the same way as in the main analysis (table S3 in supplementary materials).

**Discussion**

The main finding of this study is that lower cardiorespiratory fitness and lower muscle strength in young adulthood is predictive of severe illness and death due to COVID-19 15 to 52 years later. The protective association between cardiovascular fitness and COVID-19 severe enough to warrant hospitalization, intensive care and/or death was not attenuated by controlling for BMI at the time of conscription examinations. It was also independent of parental education and blood pressure at conscription.

As of this date no other comparable study has been published on the link between fitness in young adulthood and later COVID-19. Using data from the Swedish military conscript registry provided us with objective measures of fitness in a uniquely large sample. This together with the prospective design, the long and near-complete follow-up from well validated hospital records and the death registry provides a strong ground for drawing conclusions.

A possible mechanism for associations between CRF and severe Covid-19 could be higher cardiovascular morbidity (registered or not) in the years after conscription examination among those with low cardiorespiratory fitness. Earlier studies using the same data have shown an association between low fitness and higher risk of heart failure, diabetes type 2, stroke, hypertension, ischemic heart disease and psychiatric disorders(10, 11, 16–18). In this regard, cardiorespiratory fitness could also be a marker for a better long-term lifestyle (less smoking, better diet).

Alternatively, there could be a direct anti-inflammatory effect or a modulation of the immune system, either acquired during adolescence and early adulthood and retained during the years leading up to 2020, or continually reinforced thanks to a habit of physical activity established early in life(20). However, protective associations with CRF were also seen when individuals with CVD during follow-up were excluded from the analysis pointing to a genuine effect of physical fitness acting via a modulation of the immune system. Of course, we cannot rule out a genetic predisposition to lower inflammatory levels, in conjunction with genetically higher cardiorespiratory fitness(21).

Despite a number of strengths, the study is not without limitations, including the fact that a large number of Swedish citizens have immigrated as adults and never went through the conscription examination. Immigrants have been overrepresented in the Swedish COVID-19 mortality and morbidity statistics with explanations ranging from crowded living conditions, lack of official information, and occupations where working from home is not an option. This group has a lower socioeconomic status and higher degree of lifestyle related risk factors (more obesity, more smoking, and more diabetes). Importantly, this group has also lower cardiorespiratory fitness, potentially contributing to explaining their higher risk(15).



For peer review only - http://bmjopen.bmj.com/site/about/guidelines.xhtml

Moreover, the results from this cohort can only be generalized to men, since the conscription process was not mandatory for women. Other limitations include some protocol changes in strength and CRF measurements over the years. For instance, a rather large number of conscripts are lacking CRF scores, predominantly during the last decade where the lowest scores were not recorded. This makes it difficult to draw conclusions regarding very low CRF levels and could underestimate the adverse association. In contrast to the CRF scores the muscular strength measurements among the conscripts are normally distributed, and the analysis shows a considerable increase in the odds of developing severe COVID-19 among those at the lowest end of the distribution. When cardiorespiratory fitness was included in the model the association was weakened, indicating that CRF is the more important factor.

A related concern is the current age-range of the cohort (32-70 years in 2020). All reported findings were adjusted for exact age at conscription and year of conscription examination, and thus indirectly for age in 2020. To further illustrate this point, we repeated the analysis after stratification at median current age (52 years) and found only minor differences. This underscores that protective associations of higher fitness are observed independent of cohort differences in current age or length of follow-up.

The present study has clear clinical implications. Physical fitness is a (largely) modifiable exposure and is therefore an important preventive measure both affecting later cardiovascular disease - and as this study shows - severe COVID-19. The obvious public health implications ought to be to further strengthen efforts concerning fitness in the young and presumably through the life course. Anecdotal stories about severely sick athletes during 2020 (the first patient in intensive care in Italy was a marathon runner) raised the question if extremely physically fit individuals had a higher risk of severe COVID-19 but no statistically significant difference could be seen between those of very high cardiorespiratory fitness (score 9) and those with moderately-high (score 8), although the power of this analysis was low.

Considering the fact that women seem to run lower risk for severe COVID-19(22) it is unfortunate not to be able to compare the effects of cardiorespiratory fitness as a protective factor. Using other sources of data such as the Swedish medical birth register, this might be done in a future study. Also when data become available on later waves of the pandemic, it will be interesting to study the effects of morbidity during the years between conscription and the outcomes.

**Conclusion**

There is growing evidence of the importance of cardiorespiratory fitness on morbidity later in life(23), whereas this study shows fitness at a young age may influence the severity level of COVID-19 many years later. Later updates of the data will make it possible to understand these effects in more detail, but are unlikely to reverse the results.

The findings of the present study reinforce the need to promote regular physical activity to increase the general cardiorespiratory fitness and muscular strength of the population, not only to decrease the risk of



For peer review only - http://bmjopen.bmj.com/site/about/guidelines.xhtml

future cardiovascular events and other conditions but also to offer protection against potential consequences of coming viral pandemics.

**Captions for figures and tables**

**Figure 1**: Construction of analytic sample

**Figure 2**: Association between categories of cardiorespiratory fitness (CRF) and severe COVID-19 (left panel), and between muscle strength as a continuous predictor and severe COVID-19 (right panel, OR per SD of muscle strength). All regression models were adjusted for year and place, as well as age and BMI at conscription examination.

[All numbers for this figure can be found in tables S1 and S2]

**Author contributions:** LL and MÅ initiated the project. AG and KM performed all statistical analyses. AG had main responsibility for writing the article. MB, JR, JN, MA and AR contributed to the structure and content of the manuscript, all authors have read and approved of the final draft.

**Word count**: 3 112

**Data sharing statement**: The data used in this study is available on request from the Swedish National Board of Health and Welfare, the Swedish intensive care registry and the Swedish tax agency.

**Copyright statement:** " I, the Submitting Author has the right to grant and does grant on behalf of all authors of the Work (as defined in the below author licence), an exclusive licence and/or a non-exclusive licence for contributions from authors who are: i) UK Crown employees; ii) where BMJ has agreed a CC-BY licence shall apply, and/or iii) in accordance with the terms applicable for US Federal Government officers or employees acting as part of their official duties; on a worldwide, perpetual, irrevocable, royalty-free basis to BMJ Publishing Group Ltd ("BMJ") its licensees and where the relevant Journal is co-owned by BMJ to the co-owners of the Journal, to publish the Work in BMJ Open and any other BMJ products and to exploit all rights, as set out in our licence.

The Submitting Author accepts and understands that any supply made under these terms is made by BMJ to the Submitting Author unless you are acting as an employee on behalf of your employer or a postgraduate student of an affiliated institution which is paying any applicable article publishing charge ("APC") for Open Access articles. Where the Submitting Author wishes to make the Work available on



For peer review only - http://bmjopen.bmj.com/site/about/guidelines.xhtml

an Open Access basis (and intends to pay the relevant APC), the terms of reuse of such Open Access shall be governed by a Creative Commons licence – details of these licences and which Creative Commons licence will apply to this Work are set out in our licence referred to above."

**References**

1. Clark A, Jit M, Warren-Gash C, Guthrie B, Wang HH, Mercer SW, et al. Global, regional, and national estimates of the population at increased risk of severe COVID-19 due to underlying health conditions in 2020: a modelling study. Lancet Glob Health. 2020;8(8):e1003–17.

2. Ross R, Blair S, Arena R, Church T, Després J, Franklin B, et al. American Heart Association Physical Activity Committee of the Council on Lifestyle and Cardiometabolic Health; Council on Clinical Cardiology; Council on Epidemiology and Prevention; Council on Cardiovascular and Stroke Nursing; Council on Functional Genomics and Translational Biology; Stroke Council. Importance of assessing cardiorespiratory fitness in clinical practice: a case for fitness as a clinical vital sign: a scientific statement from the American Heart Association. Circulation. 2016;134(24):e653–99.

3. Zbinden-Foncea H, Francaux M, Deldicque L, Hawley JA. Does high cardiorespiratory fitness confer some protection against pro-inflammatory responses after infection by SARS-CoV-2? Obesity. 2020;

4. Yates T, Razieh C, Zaccardi F, Seidu S, Davies MJ, Khunti K. Obesity, walking pace and risk of severe COVID-19: Analysis of UK Biobank. medRxiv. 2020 Jan 1;2020.07.10.20150003.

5. Åberg MA, Pedersen NL, Torén K, Svartengren M, Bäckstrand B, Johnsson T, et al. Cardiovascular fitness is associated with cognition in young adulthood. Proc Natl Acad Sci. 2009;106(49):20906–11.

6. Prince SA, Adamo KB, Hamel ME, Hardt J, Gorber SC, Tremblay M. A comparison of direct versus self-report measures for assessing physical activity in adults: a systematic review. Int J Behav Nutr Phys Act. 2008;5(1):1–24.

7. Berg C, Rosengren A, Aires N, Lappas G, Toren K, Thelle D, et al. Trends in overweight and obesity from 1985 to 2002 in Göteborg, West Sweden. Int J Obes. 2005;29(8):916–24.

8. Nordesjö L, Schéle R. Validity of an ergometer cycle test and measures of isometric muscle strength when prediction some aspects of military performance. Swed J Def Med. 1974;10:11–23.

9. Lissner L, Mehlig K, Rosengren A, Torén K, Åberg MAI. A Growing Social Divide in Body Mass Index, Strength, and Fitness of Swedish Male Conscripts. J Adolesc Health 2019 Vol 65 Iss 2 Pp 232-238. 2019;65(2):232–8.

10. Åberg ND, Kuhn HG, Nyberg J, Waern M, Friberg P, Svensson J, et al. Influence of cardiovascular fitness and muscle strength in early adulthood on long-term risk of stroke in Swedish men. Stroke. 2015;46(7):1769–76.

11. World Health Organization. The Asia-Pacific perspective: redefining obesity and its treatment. 2000;



For peer review only - http://bmjopen.bmj.com/site/about/guidelines.xhtml

12. Cole TJ, Lobstein T. Extended international (IOTF) body mass index cut-offs for thinness, overweight and obesity. Pediatr Obes. 2012;7(4):284–94.

13. Williams B, Mancia G, Spiering W, Agabiti Rosei E, Azizi M, Burnier M, et al. 2018 ESC/ESH Guidelines for the management of arterial hypertension: The Task Force for the management of arterial hypertension of the European Society of Cardiology (ESC) and the European Society of Hypertension (ESH). Eur Heart J. 2018;39(33):3021–104.

14. Li R, Hertzmark E, Louie M, Chen L, Spiegelman D. The sas lgtphcurv9 macro. Boston Channing Lab. 2011;

15. Lindgren M, Börjesson M, Ekblom Ö, Bergström G, Lappas G, Rosengren A. Physical activity pattern, cardiorespiratory fitness, and socioeconomic status in the SCAPIS pilot trial—a cross-sectional study. Prev Med Rep. 2016;4:44–9.

16. Lindgren M, Åberg M, Schaufelberger M, Åberg D, Schiöler L, Torén K, et al. Cardiorespiratory fitness and muscle strength in late adolescence and long-term risk of early heart failure in Swedish men. Eur J Prev Cardiol. 2017;24(8):876–84.

17. Crump C, Sundquist J, Winkleby MA, Sundquist K. Interactive effects of obesity and physical fitness on risk of ischemic heart disease. Int J Obes. 2017;41(2):255–61.

18. Crump C, Sundquist J, Winkleby MA, Sundquist K. Interactive effects of physical fitness and body mass index on the risk of hypertension. JAMA Intern Med. 2016;176(2):210–6.

19. Crump C, Sundquist J, Winkleby MA, Sieh W, Sundquist K. Physical fitness among Swedish military conscripts and long-term risk for type 2 diabetes mellitus: a cohort study. Ann Intern Med. 2016;164(9):577–84.

20. Gleeson M, Bishop NC, Stensel DJ, Lindley MR, Mastana SS, Nimmo MA. The anti-inflammatory effects of exercise: mechanisms and implications for the prevention and treatment of disease. Nat Rev Immunol. 2011;11(9):607–15.

21. Burtscher J, Millet GP, Burtscher M. Low cardiorespiratory and mitochondrial fitness as risk factors in viral infections: implications for COVID-19. 2020;

22. Peckham H, de Gruijter NM, Raine C, Radziszewska A, Ciurtin C, Wedderburn LR, et al. Male sex identified by global COVID-19 meta-analysis as a risk factor for death and ITU admission. Nat Commun. 2020;11(1):1–10.

23. Ekblom-Bak E, Ekblom B, Söderling J, Börjesson M, Blom V, Kallings LV, et al. Sex-and age-specific associations between cardiorespiratory fitness, CVD morbidity and all-cause mortality in 266.109 adults. Prev Med. 2019;127:105799.

For peer review only

For peer review only

15

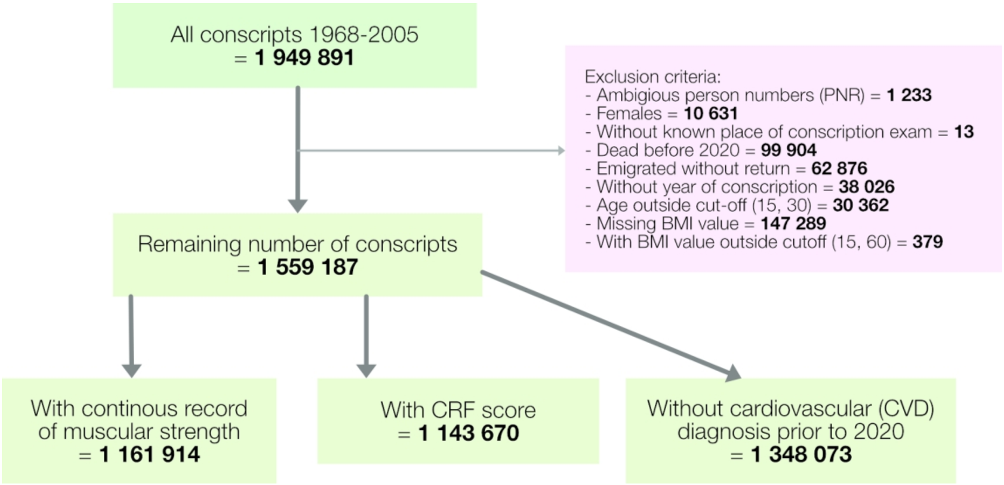


Figure 1: Construction of analytic sample

154x73mm (300 x 300 DPI)

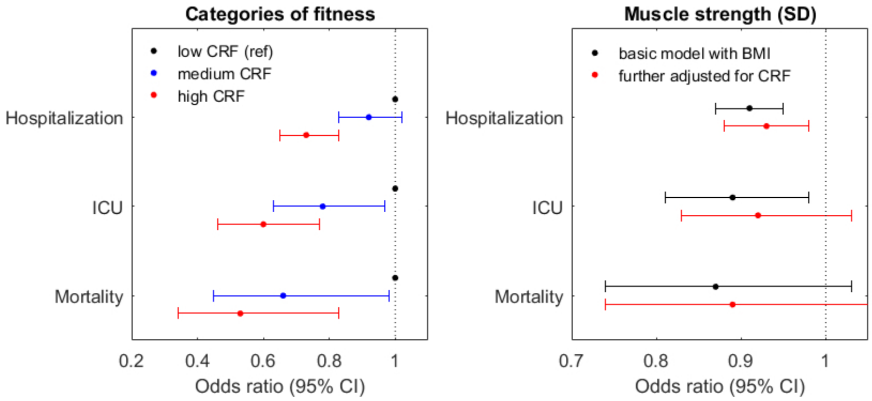


Figure 2: Association between categories of cardiorespiratory fitness (CRF) and severe COVID-19 (left panel), and between muscle strength as a continuous predictor and severe COVID-19 (right panel, OR per SD of muscle strength). All regression models were adjusted for year and place, as well as age and BMI at conscription examination.

247x98mm (96 x 96 DPI)

Supplementary material

**Table S1:** Results of main analysis. Association between conscript's fitness at time of examination and hospitalization, intensive care and death due to COVID-19 (OR, 95% CI)

| Fitness level | Basic model [1] n=1 143 670 | Basic model controlled for BMI [2] n=1 143 670 | Basic model controlled for blood pressure subgroup n=1 140 514 | Basic model controlled for parental education (low, medium, high) n=1 031 246 | Basic model controlled for BMI category, blood pressure and parental education n=1 028 122 |
|---|---|---|---|---|---|
| **All hospitalization due to COVID-19.** | | | | | |
| | cases=2 006 | cases=2 006 | cases=2 003 | cases=1 710 | cases=1 707 |
| Low (1-5) | **1** | **1** | **1** | **1** | **1** |
| Medium (6-7) | 0.90 (0.80-1.00) | 0.92 (0.83-1.02) | 0.91 (0.82-1.01) | 0.91 (0.82-1.02) | 0.92 (0.82-1.03) |
| High (8-9) | **0.75** (0.66-0.85) | **0.73** (0.65-0.83) | **0.76** (0.68-0.85) | **0.77** (0.68-0.88) | **0.75** (0.66-0.85) |
| AUROC | 0.66 | 0.66 | 0.66 | 0.66 | 0.67 |
| **Intensive care due to COVID-19** | | | | | |
| | cases=445 | cases=445 | cases=445 | cases=363 | cases=363 |
| Low (1-5) | **1** | **1** | **1** | **1** | **1** |
| Medium (6-7) | **0.75** (0.59-0.95) | **0.78** (0.63-0.97) | **0.76** (0.62-0.95) | **0.76** (0.60-0.97) | **0.78** (0.61-0.99) |
| High (8-9) | **0.62** (0.48-0.82) | **0.60** (0.46-0.77) | **0.61** (0.48-0.78) | **0.65** (0.49-0.85) | **0.62** (0.47-0.82) |
| AUROC | 0.69 | 0.71 | 0.70 | 0.70 | 0.72 |
| **Death due to COVID-19** | | | | | |
| | cases=149 | cases=149 | cases=149 | cases= 111 | cases=111 |
| Low (1-5) | **1** | **1** | **1** | **1** | **1** |
| Medium (6-7) | **0.66** (0.45-0.97) | **0.66** (0.45-0.98) | **0.67** (0.46-0.98) | **0.55** (0.35-0.86) | **0.55** (0.35-0.88) |
| High (8-9) | **0.56** (0.37-0.85) | **0.53** (0.34-0.83) | **0.57** (0.37-0.87) | **0.56** (0.35-0.90) | **0.53** (0.33-0.88) |
| AUROC | 0.81 | 0.82 | 0.81 | 0.81 | 0.83 |

[1] Adjusted for year and place of examination, and age at examination.

[2] BMI, $BMI^2$, $BMI^3$

For peer review only

**Figure S1**: Results of linear and cubic spline analysis of continuous muscle strength and severity of COVID-19.

**Table S2:** Association between muscle strength at conscription and severity of COVID disease

| Endpoint | Total | # cases | OR[c] | Auroc |
|---|---|---|---|---|
| Hospital[a] | 1161827 | 2252 | **0.91***** (0.87-0.95) | 0.64 |
| ICU[a] | | 514 | **0.89*** (0.81-0.98) | 0.68 |
| Mortality[a] | | 180 | 0.87 (0.74-1.03) | 0.80 |
| | | | | |
| Hospital[b] | 952225 | 1850 | **0.93**** (0.88-0.98) | 0.64 |
| ICU[b] | | 421 | 0.92 (0.83-1.03) | 0.68 |
| Mortality[b] | | 146 | 0.89 (0.74-1.07) | 0.80 |

[a] basic model adjusted for BMI, $BMI^2$, $BMI^3$

[b] above model further adjusted for fitness categories

[c] OR for disease by 1 SD higher muscle strength (SD = 313 N)

**Table S3**: Results of sensitivity analysis. Association between conscript's fitness and muscle strength at time of examination and hospitalization, intensive care and death due to COVID-19 (OR, 95% CI). All incident cases of CVD excluded.

| | **Hospitalization due to COVID-19 (n=1 334)** | **Intensive care due to COVID-19 (n=301)** | **Death due to COVID-19 (n=69)** |
|---|---|---|---|
| **Fitness level** | Basic model[1] adjusted for BMI, $BMI^2$, $BMI^3$ | | |
| Low (1-5) | **1** | **1** | **1** |
| Medium (6-7) | 0.98 (0.86-1.11) | 0.90 (0.69-1.17) | 0.79 (0.47-1.34) |
| High (8-9) | **0.74** (0.64-0.86) | **0.63** (0.45-0.86) | **0.33** (0.16-0.72) |
| AUROC | 0.65 | 0.70 | 0.79 |
| **Muscle strength per 1 SD increase** | | | |
| OR | **0.93** (0.88-0.98) | **0.86** (0.77-0.97) | 0.83 (0.66-1.04) |

For peer review only

| | | | |
|---|---|---|---|
| OR when further adjusted for fitness | 0.96 (0.90-1.02) | 0.91 (0.80-1.04) | 0.90 (0.69-1.18) |

[1] Adjusted for year and place of examination, age at examination.

**Table S4.** Included main diagnoses divided by categories when COVID-19 is secondary diagnosis.

| Included categories | ICD-codes |
|---|---|
| **COVID-related symptoms** | |
| Cough | R05 |
| Abnormalities of breathing | R06 |
| Pain in throat and chest | R07 |
| Other symptoms and signs involving the circulatory and respiratory system | R09 |
| Dizziness and giddiness | R42 |
| Fever of other or unknown origin | R50 |
| Headache | R51 |
| Malaise and fatigue | R53 |
| Syncope and collapse | R55 |
| **Upper and lower respiratory tract infections** | |
| Acute nasopharyngitis | J00 |
| Acute tonsillitis | J03 |
| Acute upper respiratory infections of multiple and unspecified sites | J06 |
| Influenza due to other identified influenza virus | J10 |
| Other viral pneumonia | J128 |
| Viral pneumonia, unspecified | J129 |
| Pneumonia due to Streptococcus pneumoniae | J13 |
| Bacterial pneumonia, not elsewhere classified | J15 |
| Pneumonia due to other specified infectious organisms | J168 |

For peer review only

| | |
|---|---|
| Pneumonia in diseases classified elsewhere | J17 |
| Pneumonia, unspecified organism | J18 |
| Unspecified acute lower respiratory infection | J22 |
| Coronavirus infection, unspecified | B342 |
| Other viral infections of unspecified site | B348 |
| Viral infection, unspecified | B349 |
| Coronavirus as the cause of diseases classified elsewhere | B972 |
| Other and unspecified infectious diseases | B99 |
| **Respiratory disorders** | |
| Pulmonary embolism | I26 |
| Acute respiratory distress syndrome | J80 |
| Pulmonary edema | J81 |
| Pleural effusion not elsewhere classified | J90 |
| Respiratory failure, not elsewhere classified | J96 |
| Respiratory disorders in diseases classified elsewhere | J99 |
| **Obstructive Airway Diseases** | |
| Acute bronchitis due to other specified organisms | J208 |
| Acute bronchitis, unspecified | J209 |
| Acute bronchiolitis due to other specified organisms | J218 |
| Acute bronchiolitis, unspecified | J219 |
| Other chronic obstructive pulmonary disease | J44 |
| Asthma | J45 |
| **Cardiac diseases** | |
| Viral carditis | B332 |
| Chronic ischemic heart disease | I25 |
| Acute pericarditis | I30 |
| Pericarditis in diseases classified elsewhere | I32 |

| | |
|---|---|
| Acute myocarditis, unspecified | I40 |
| Myocarditis in diseases classified elsewhere | I41 |
| Atrial fibrillation and flutter | I48 |
| Heart failure | I50 |
| Abnormalities of heart beat | R00 |
| **Kidney disorders** | |
| Acute kidney failure | N17 |
| Chronic kidney disease | N18 |
| Unspecified kidney failure | N19 |
| **Electrolyte disorders** | |
| Other disorders of fluid, electrolyte and acid-base balance | E87 |

For peer review only

STROBE Statement—Checklist of items that should be included in reports of ***cohort studies***

| | Item No | Recommendation | Page No |
|---|---|---|---|
| **Title and abstract** | 1 | (*a*) Indicate the study's design with a commonly used term in the title or the abstract<br>(*b*) Provide in the abstract an informative and balanced summary of what was done and what was found | 4 |
| **Introduction** | | | |
| Background/rationale | 2 | Explain the scientific background and rationale for the investigation being reported | 5 |
| Objectives | 3 | State specific objectives, including any prespecified hypotheses | 5 |
| **Methods** | | | |
| Study design | 4 | Present key elements of study design early in the paper | 5 |
| Setting | 5 | Describe the setting, locations, and relevant dates, including periods of recruitment, exposure, follow-up, and data collection | 6 |
| Participants | 6 | (*a*) Give the eligibility criteria, and the sources and methods of selection of participants. Describe methods of follow-up<br>(*b*) For matched studies, give matching criteria and number of exposed and unexposed | 7 |
| Variables | 7 | Clearly define all outcomes, exposures, predictors, potential confounders, and effect modifiers. Give diagnostic criteria, if applicable | 7-8 |
| Data sources/ measurement | 8* | For each variable of interest, give sources of data and details of methods of assessment (measurement). Describe comparability of assessment methods if there is more than one group | 5-6 |
| Bias | 9 | Describe any efforts to address potential sources of bias | 5 |
| Study size | 10 | Explain how the study size was arrived at | 7 |
| Quantitative variables | 11 | Explain how quantitative variables were handled in the analyses. If applicable, describe which groupings were chosen and why | 6-7 |
| Statistical methods | 12 | (*a*) Describe all statistical methods, including those used to control for confounding<br>(*b*) Describe any methods used to examine subgroups and interactions<br>(*c*) Explain how missing data were addressed<br>(*d*) If applicable, explain how loss to follow-up was addressed<br>(*e*) Describe any sensitivity analyses | 8 |
| **Results** | | | |
| Participants | 13* | (a) Report numbers of individuals at each stage of study—eg numbers potentially eligible, examined for eligibility, confirmed eligible, included in the study, completing follow-up, and analysed<br>(b) Give reasons for non-participation at each stage<br>(c) Consider use of a flow diagram | 7, 9 |
| Descriptive data | 14* | (a) Give characteristics of study participants (eg demographic, clinical, social) and information on exposures and potential confounders<br>(b) Indicate number of participants with missing data for each variable of interest<br>(c) Summarise follow-up time (eg, average and total amount) | 9 |
| Outcome data | 15* | Report numbers of outcome events or summary measures over time | 9 |

For peer review only



For peer review only - http://bmjopen.bmj.com/site/about/guidelines.xhtml

| | | | |
|---|---|---|---|
| Main results | 16 | (*a*) Give unadjusted estimates and, if applicable, confounder-adjusted estimates and their precision (eg, 95% confidence interval). Make clear which confounders were adjusted for and why they were included<br>(*b*) Report category boundaries when continuous variables were categorized<br>(*c*) If relevant, consider translating estimates of relative risk into absolute risk for a meaningful time period | 9 |
| Other analyses | 17 | Report other analyses done—eg analyses of subgroups and interactions, and sensitivity analyses | 9-10 |
| **Discussion** | | | |
| Key results | 18 | Summarise key results with reference to study objectives | 10 |
| Limitations | 19 | Discuss limitations of the study, taking into account sources of potential bias or imprecision. Discuss both direction and magnitude of any potential bias | 10 |
| Interpretation | 20 | Give a cautious overall interpretation of results considering objectives, limitations, multiplicity of analyses, results from similar studies, and other relevant evidence | 10 |
| Generalisability | 21 | Discuss the generalisability (external validity) of the study results | 10 |
| **Other information** | | | |
| Funding | 22 | Give the source of funding and the role of the funders for the present study and, if applicable, for the original study on which the present article is based | 4 |

*Give information separately for exposed and unexposed groups.

**Note:** An Explanation and Elaboration article discusses each checklist item and gives methodological background and published examples of transparent reporting. The STROBE checklist is best used in conjunction with this article (freely available on the Web sites of PLoS Medicine at http://www.plosmedicine.org/, Annals of Internal Medicine at http://www.annals.org/, and Epidemiology at http://www.epidem.com/). Information on the STROBE Initiative is available at http://www.strobe-statement.org.

For peer review only

BMJ Open

# Fitness, strength and severity of COVID-19 – a prospective register study on 1 559 187 Swedish conscripts.



| | |
|---|---|
| Journal: | *BMJ Open* |
| Manuscript ID | bmjopen-2021-051316.R1 |
| Article Type: | Original research |
| Date Submitted by the Author: | 25-May-2021 |



| | |
|---|---|
| Complete List of Authors: | af Geijerstam, Agnes; University of Gothenburg Sahlgrenska Academy, School of public health and community medicine, department of medicine<br>Mehlig, Kirsten; University of Gothenburg Sahlgrenska Academy, School of Public Health and Community Medicine<br>Börjesson, Mats; University of Gothenburg Sahlgrenska Academy, Dept of Molecular and Clinical Medicine; University of Gothenburg, Center for Health and Performance<br>Robertson, Josefina; University of Gothenburg Sahlgrenska Academy, School of Public Health and Community Medicine; University of Gothenburg Sahlgrenska Academy, Department of Infectious Diseases, Institute of Biomedicine<br>Nyberg, Jenny; University of Gothenburg Sahlgrenska Academy, Section for Clinical Neuroscience, Institute of Neuroscience and Physiology<br>Adiels, Martin; University of Gothenburg Sahlgrenska Academy, School of Public health and Community Medicine; University of Gothenburg Sahlgrenska Academy, Dept of Molecular and Clinical Medicine<br>Rosengren, Annika ; University of Gothenburg Sahlgrenska Academy, Department of Molecular and Clinical Medicine; Sahlgrenska University Hospital<br>Åberg, Maria; University of Gothenburg Sahlgrenska Academy, a School of Public Health and Community Medicine, Institute of Medicine; Region Västra Götaland, Regionhälsan<br>Lissner, Lauren; University of Gothenburg Sahlgrenska Academy, School of Public Health and Community Medicine, Institute of Medicine |



| | |
|---|---|
| <b>Primary Subject Heading</b>: | Epidemiology |
| Secondary Subject Heading: | Infectious diseases, Public health |
| Keywords: | COVID-19, EPIDEMIOLOGY, Public health < INFECTIOUS DISEASES |
| | |


SCHOLARONE™ Manuscripts

*I, the Submitting Author has the right to grant and does grant on behalf of all authors of the Work (as defined in the below author licence), an exclusive licence and/or a non-exclusive licence for contributions from authors who are: i) UK Crown employees; ii) where BMJ has agreed a CC-BY licence shall apply, and/or iii) in accordance with the terms applicable for US Federal Government officers or employees acting as part of their official duties; on a worldwide, perpetual, irrevocable, royalty-free basis to BMJ Publishing Group Ltd ("BMJ") its licensees and where the relevant Journal is co-owned by BMJ to the co-owners of the Journal, to publish the Work in this journal and any other BMJ products and to exploit all rights, as set out in our licence.*

*The Submitting Author accepts and understands that any supply made under these terms is made by BMJ to the Submitting Author unless you are acting as an employee on behalf of your employer or a postgraduate student of an affiliated institution which is paying any applicable article publishing charge ("APC") for Open Access articles. Where the Submitting Author wishes to make the Work available on an Open Access basis (and intends to pay the relevant APC), the terms of reuse of such Open Access shall be governed by a Creative Commons licence – details of these licences and which Creative Commons licence will apply to this Work are set out in our licence referred to above.*

*Other than as permitted in any relevant BMJ Author's Self Archiving Policies, I confirm this Work has not been accepted for publication elsewhere, is not being considered for publication elsewhere and does not duplicate material already published. I confirm all authors consent to publication of this Work and authorise the granting of this licence.*

For peer review only

# Fitness, strength and severity of COVID-19 – a prospective register study of 1 559 187 Swedish conscripts.

Authors: Agnes af Geijerstam [a], Kirsten Mehlig [a], Mats Börjesson [b, c ,d], Josefina Robertson [a, d, e,], Jenny Nyberg [f,], Martin Adiels [a, b] , Annika Rosengren [b, d], Maria Åberg [a, g], Lauren Lissner [a]

[a] School of Public Health and Community Medicine, Institute of Medicine, Sahlgrenska Academy, University of Gothenburg, Gothenburg, Sweden

[b] Dept of Molecular and Clinical Medicine, Sahlgrenska Academy, University of Gothenburg, Gothenburg, Sweden

[c] Center for Health and Performance, University of Gothenburg, Gothenburg, Sweden

[d] Sahlgrenska University Hospital, Region Västra Götaland, Gothenburg, Sweden

[e] Department of Infectious Diseases, Institute of Biomedicine, Sahlgrenska Academy, University of Gothenburg, Gothenburg, Sweden

[f] Section for Clinical Neuroscience, Institute of Neuroscience and Physiology, Sahlgrenska Academy, University of Gothenburg, Gothenburg, Sweden

[g] Region Västra Götaland, Regionhälsan, Gothenburg, Sweden

For peer review only

**Contributor statement:** LL and MÅ initiated the project. AG and KM performed all statistical analyses. AG had main responsibility for writing the article. MB, JR, JN, MA and AR all made substantial contributions to the interpretation of the analyses, the structure and content of the manuscript, and have read and approved of the final draft. All authors have agreed to be accountable for all aspects of the work.

**Corresponding author:** Agnes af Geijerstam, School of Public Health and Community Medicine, Institute of Medicine, Sahlgrenska Academy, University of Gothenburg, P.O Box 459, 405 30 Gothenburg, Sweden. agnes.af.geijerstam@gu.se

**Funding statement:** This work was supported by the EpiLife-Teens Research Program (FORMAS-2012-00038), by the Swedish ALF-agreement (ALFGBG-720201) and the Swedish Research Council (02508, VRREG 2019-00193, 2020-05792). The funding sources had no role in study design, collection, analysis and interpretation of data, the writing of the report, or the decision to submit the article for publication.

**Competing interest statement**: All authors have completed the Unified Competing Interest form (available on request from the corresponding author) and declare: no support from any organization for the submitted work; no financial relationships with any organizations that might have an interest in the submitted work in the previous three years, no other relationships or activities that could appear to have influenced the submitted work.



For peer review only - http://bmjopen.bmj.com/site/about/guidelines.xhtml

**Transparency statement:** AG affirms that the manuscript is an honest, accurate, and transparent account of the study being reported and that no important aspects of the study have been omitted.

**Dissemination to participants and related patient and public communities:** We will make the findings of this article public with a press release and via media contacts.

**Keywords:** COVID-19, physical fitness, cardiorespiratory fitness, muscular strength, adolescence, men.

For peer review only



For peer review only - http://bmjopen.bmj.com/site/about/guidelines.xhtml

## Abstract

**Objective** To investigate the possible connection between cardiorespiratory fitness and muscle strength in early adulthood and severity of COVID-19 later in life.

**Design** Prospective registry based cohort study.

**Participants** 1 559 187 Swedish men, undergoing military conscription between 1968 and 2005 at a mean age of 18.3 (SD 0.73) years.

**Main outcome measures** Hospitalization, intensive care or death due to COVID-19 from March to September 2020, in relation to cardiorespiratory fitness (CRF) and muscle strength.

**Results** High cardiorespiratory fitness in late adolescence and early adulthood had a protective association with severe COVID-19 later in life with OR (95% CI) 0.76 (0.67-0.85) for hospitalization (n=2 006), 0.61 (0.48-0.78) for intensive care (n=445), and 0.56 (0.37-0.85) for mortality (n=149), compared to the lowest category of CRF. The association remains unchanged when controlled for body mass index (BMI), blood pressure, chronic diseases and parental education level at baseline, and incident cardiovascular disease before 2020. Moreover lower muscle strength in late adolescence showed a linear association with a higher risk of all three outcomes when controlled for BMI and height.

**Conclusions** Physical fitness at a young age is associated with severity of COVID-19 many years later. This underlines the necessity to increase the general physical fitness of the population to offer protection against coming viral pandemics.


## Strengths and limitations of this study

# Data from the Swedish military conscript registry provided us with objective measures of fitness in a uniquely large sample.

# The prospective design with long and near-complete follow-up from well validated hospital records and the death registry gives a strong ground for drawing conclusions.

# Limitations include that the results from this cohort can only be generalized to men, since the conscription process was not mandatory for women.

# During the last decade the lowest cardiorespiratory fitness scores were not recorded in the registry, making it difficult to draw conclusions regarding very low CRF, and maybe underestimating the associations.



For peer review only

For peer review only - http://bmjopen.bmj.com/site/about/guidelines.xhtml

**Background**

The COVID-19 pandemic has affected the world in unprecedented ways. While measures such as contact restrictions and vaccinations relieve the immediate impact of the disease, epidemiologic studies may identify risk factors that could be managed in the long run, in order to reduce the impact of future epidemics. Early on, advanced age and current cardiovascular comorbidities including obesity were found to be associated with a more severe course of coronavirus disease(1–3).

Cardiorespiratory fitness is regarded as one of the most important risk factors for overall health, cardiovascular morbidity and overall mortality(4). The level of physical activity early in life (and associated cardiorespiratory fitness) may have short and long-term effects on risk factors and the immune system. There has been speculation about the way it may confer protection against severe COVID-19(5). An early observation from the UK Biobank cohort that slower self-reported walking pace predicted more severe COVID-19 suggests the importance of cardiorespiratory fitness for this particular disease(6), and more recent studies have reinforced this notion, using self-reported measures(7,8) but also clinically tested cardiorespiratory fitness and strength(9,10).

Although cardiorespiratory fitness is a persistent trait with a genetic component(11), it is also to a large extent modifiable by regular exercise. Overall, physical fitness consists of cardiorespiratory fitness and muscular fitness, including muscle strength and healthy weight. BMI is routinely measured in hospital and public care settings and has already been shown to be associated with severity of disease(2,12). However, objective measures of cardiorespiratory fitness and strength before the onset of COVID-19 is rarely available in population based studies. Self-reported measures are unreliable and most studies conducted in middle-age or older populations have limited information on risk factors in earlier life(13). Participation rates among young men in population-based studies also tend to be low(14).

The Swedish Military Conscripts registry contains detailed and high-quality information about cardiorespiratory fitness and strength in a uniquely large sample of young men. In contrast to many population-based surveys, the participation rates until the early 2000's yielded highly generalizable results. Combined with measured data on BMI and other potential confounding factors in early adulthood, linkage data from nation-wide disease registries enables a life-course perspective on the role of physical fitness as a potentially mitigating factor for severe COVID-19 that is presently lacking in the literature.

**Methods**

**Study design**

This prospective cohort study is based on data from the Swedish military conscript registry, the longitudinal integration database for health insurance and labor market studies (LISA registry), and the Swedish national hospital, intensive care and cause of death registries (for description in detail, see Åberg



For peer review only - http://bmjopen.bmj.com/site/about/guidelines.xhtml

et al, 2015). Fitness data was collected in early adulthood, in relation to incidence data on COVID-19 retrieved in September 2020, after the first wave of the pandemic in Sweden.

*Patient and public involvement statement*

As this is a registry based study, there have been no patient or public involvement.

*Population studied*

The Swedish military conscript's registry contains information about 1 949 891 Swedish individuals who enlisted for military service between late 1968 and 2005 giving between 15-52 years of follow-up until the outbreak of COVID-19. During that time, Swedish law required all male citizens to enlist, except for those in prison or those with severe chronic somatic or psychiatric conditions or functional disabilities (approximately 2-3% annually). Due to a shift to voluntary recruitment in 2005 the data in later years is no longer considered representative. The standardized protocol included measurements of weight and height, blood pressure, muscular strength and cardiorespiratory fitness. The registry also includes prior medical diagnoses. The examinations took place over two days in six conscription centers across Sweden.

**Main independent variables**

*Cardiorespiratory fitness score*

To evaluate cardiorespiratory fitness (CRF) the subjects performed a cycle ergometric test where the work rate was successively increased until limited by exhaustion. Based on the final work rate, a 9-level score derived by the National Service Administration in Sweden(15) served as a standardized measure of CRF. These scores were validated in relation to different tasks the conscripts were expected to manage during their time in the military (cross country running with a heavy backpack, heavy lifting)(15). Over the decades there have been some changes in the conscription examination protocols, and raw data were not available for all conscripts(16). However, the CRF scores matching certain military requirements were assigned during all recruitment years, and can be assumed to be comparable across years(11). For this study, the scores were collapsed into three categories describing low (1-5), medium (6-7) and high (8-9) CRF. The asymmetrical assignment of CRF scores was prompted by the fact that the fitness data in the lowest categories were omitted from the registry after 1999(17).

*Muscle strength score*

Isometric muscle strength in units of Newton was assessed as a weighted sum of knee extension (weight 1.3), elbow flexion (weight 0.8) and handgrip (weight 1.7). As the methods for measuring muscle strength did not change over the years and both the weighted values and assigned strength scores were available in the registry until 1996, it was possible to examine strength as a continuous variable(15).

**Other covariates**



For peer review only - http://bmjopen.bmj.com/site/about/guidelines.xhtml

*BMI, height and weight status*

Weight and height were measured by standard anthropometric measurement techniques, continuous BMI values (kg/m$^2$) were calculated, and also divided into categories of underweight (BMI <18.5), normal weight (BMI 18.5-24.9), overweight (BMI 25.0-29.9), obesity (BMI 30-34.9) and obesity class 2 (BMI ≥35)(18). For those aged 15 to 17 at conscription weight status categories were adjusted based on age- and sex-specific BMI z-scores, according to the International childhood BMI cutoffs (IOTF)(19). In addition, height was included as a covariate, given its positive association with muscular strength.

*Blood pressure*

High blood pressure in early adulthood was used as an indicator of predisposition to later hypertension and other cardiovascular diseases(6). Systolic and diastolic blood pressure was measured according to a standardized protocol after five to 10 minutes of rest in the supine position. In this study, values were then categorized into five subgroups (optimal blood pressure (systolic blood pressure <120 mm Hg and diastolic blood pressure <80 mm Hg), normal blood pressure (120-129 and 80-84 mm Hg), high normal blood pressure (130-139 and 85-89 mm Hg), grade 1 hypertension (140-159 and 90-99 mm Hg), and grade 2 hypertension (≥160 and ≥100 mm Hg) based on the 2018 European guidelines(20).

*Chronic disease at baseline*

To assess the specific effect of physical activity we controlled for morbidity at baseline, using ICD-codes for respiratory disease (J00-J99), cardiovascular disease (I00-I99), diabetes (E10-14), kidney disease (N00-N29) and malignant cancers (C00-C97).

*Parental education*

Parental education was considered a proxy for socioeconomic position of the family of origin. Using data from the LISA registry, parental education was based on the highest of maternal and paternal education, and divided into three categories with low education (up to 9 years), medium education (high school diploma with ≤2 years at university) and high (≥3 years at university)(16).

**Analytic samples and subsamples**

Figure 1 describes the construction of the analytic sample. Among 1 949 891 individuals examined for conscription between 1968 and 2005, the following exclusions were made: those with ambiguous identification numbers or without known place or year of conscription, those dying or emigrating without return before 2020, and all female conscripts, leaving 1 737 217 observations. Because of the aim of relating fitness measured in adolescence and early adulthood to disease severity later in life, individuals with age at conscription outside the interval of 15 to 30 were excluded. Since BMI was an important confounder for the association between fitness and disease, the sample was further restricted excluding all men with missing or implausible BMI-values (range for inclusion set between 15 and 60). Among the



For peer review only - http://bmjopen.bmj.com/site/about/guidelines.xhtml

remaining 1 559 187 men, 1 143 670 had values for CRF and 1 161 914 had registered values for the continuous measure of muscle strength.

[Figure 1 about here]

**Outcome parameters: Hospitalization and/or death due to COVID-19**

Using the Swedish personal identification number the full sample was linked to the national hospital, the intensive care and the cause of death registries. From these, all cases between March and September 2020 with a main diagnosis of ICD U071 for test verified infection with SARS-CoV-2 and U072 for clinically diagnosed COVID-19, or deaths with U071 or U072 as an underlying cause of death were identified.

Records with U071 or U072 as a secondary diagnosis were counted as cases if the main diagnosis was clinically related to COVID-19 (full list in table S1 in supplementary materials). This resulted in a reduction of total cases from 2 957 to 2 635.

All cases were considered as severe COVID-19 since they required hospital care. Furthermore, patients were categorized into three groups with increasing severity;

(I) Hospitalization due to COVID-19, n = 2594
(II) Intensive care due to COVID-19, n = 559
(III) Deaths due to COVID-19, n = 184

Register data based on Swedish hospital records have high validity(21). As the health care system was stressed during 2020 but never overloaded, it is unlikely that a significant number of potential hospital patients with COVID-19 were not admitted or included in the records.

**Statistical analysis**

The main independent variable, cardiorespiratory fitness, was analyzed in terms of three categories describing low, medium, and high values of exposure. Logistic regression was used to calculate the odds for COVID-19 outcome by this exposure category, adjusted for regional examination center, age at and year of conscription, as well as linear, quadratic and cubic terms of BMI, height, parental education, chronic disease and blood pressure at examination. Due to the rarity of events, in particular deaths from COVID-19, we used a penalized likelihood estimation (Firth method) to reduce possible small-sample bias in maximum likelihood estimation.(22).

Effect modification was investigated by including product terms between the main exposure (CRF categories) and other categorical predictors into the model, overweight status (BMI $\geq 25$ kg/m$^2$), parental



For peer review only - http://bmjopen.bmj.com/site/about/guidelines.xhtml

education, blood pressure category, and current age dichotomized at median value (52 years). For each interaction analysis, the p-value from an F-test comparing a model with and without interactions was assessed. Results were given in terms of odds ratios (OR) and 95% confidence intervals (95% CI). The area under the receiver operating characteristics curve (AUROC) was used as a measure for the discriminative properties of the logistic models. Muscle strength in units of Newton (N) was standardized to zero mean and unit standard deviation (SD), and results were given in terms of OR per SD. To explore the non-linear association between muscle strength and COVID-19 we used logistic regression based on restricted cubic splines(23). Considering the rarity of events, the odds ratios may be interpreted as relative risk, according to the rare disease assumption.

To investigate whether the associations with CRF or strength were explained by differences in cardiovascular disease (CVD) during midlife, we conducted a mediation analysis including confounding of exposure-mediator-outcome associations with Delta method for confidence intervals.(24)

Statistical analyses were performed with SAS version 9.4 (SAS Institute, Cary, NC). Statistical significance was set at 0.05 (2-sided tests).

### Ethics approval statement

The study conforms to the principles outlined in the Declaration of Helsinki. The Ethics Committee of the University of Gothenburg and Confidentiality Clearance at Statistics Sweden approved the study (EPN Reference numbers EPN 462-14 and 567-15; T174-15, T653-17, T196-17, T 2020-01325, T 2020-02420). The requirement for informed consent was waived by the Ethics Committee of the University of Gothenburg for secondary analysis of existing data. The data was anonymised before being accessed by the study authors.

### Results

Basic characteristics of the 1 559 187 men included in the study are shown by conscription decade in table 1. The majority of deaths and hospitalizations due to COVID-19 were observed among those who underwent conscription between 1968 and 1985.

**Table 1**: Description of analytical sample, by conscription decade.

| ***Conscription year*** | **1968-1975** | **1976-1985** | **1986-1995** | **1996-2005** | **All decades** |
|---|---|---|---|---|---|
| *N* | 283 082 | 441 414 | 496 484 | 338 207 | 1 559 187 |
| *Age at conscription, mean (SD)* | 18.5 (0.65) | 18.3 (0.82) | 18.3 (0.76) | 18.2 (0.58) | 18.3 (0.73) |
| *Age in 2020 mean (SD)* | 65.9 (1.94) | 57.5 (2.98) | 48.0 (3.00) | 38.3 (2.91) | 51.8 (9.89) |
| | | | ***CRF*** | | |
| *n with CRF score* | 248 424 | 380 791 | 358 731 | 155 720 | 1 143 670 |
| *CRF low n (%)* | 89 953 (36.2) | 135 569 (35.6) | 85 203 (23.8) | 25 546 (16.4) | 336 271 (29.4) |
| *CRF medium n (%)* | 84 189 (33.9) | 127 965 (33.6) | 189 000 (52.7) | 87 343 (56.1) | 488 497 (42.7) |
| *CRF high n (%)* | 74 286 (29.9) | 117 257 (30.8) | 84 528 (23.6) | 42 831 (27.5) | 318 902 (27.9) |
| | | | ***Muscle strength*** | | |


For peer review only - http://bmjopen.bmj.com/site/about/guidelines.xhtml

| | | | | | |
|---|---|---|---|---|---|
| *n with strength score in Newton* | 277 150 | 435 32 | 449 188 | n/a | 1 161 661 |
| *Mean score (SD)* | 2 033 (299) | 2 092 (315) | 2 140 (315) | n/a | 2 096 (313) |
| | | | ***BMI*** | | |
| *Mean (SD)* | 21.2 (2.6) | 21.7 (2.8) | 22.0 (3.0) | 22.7 (3.6) | 21.9 (3.0) |
| *Overweight and obese n (%)* | 20 481 (1.5) | 44 265 (2.2) | 65 268 (3.2) | 64 943 (5.6) | 194 957 (3.6) |
| | | | ***Height*** | | |
| *Mean cm (SD)* | 178.7 (6.4) | 179.1 (6.5) | 179.3 (6.6) | 179.8 (6.7) | 179.2 (6.7) |
| | | | ***Blood pressure*** | | |
| *Grade 1 hypertension, n (%)* | 51 242 (18.1) | 81 990 (18.6) | 89 752 (18.5) | 57 582 (21.4) | 280 566 (19) |
| *Grade 2 hypertension, n (%)* | 2 167 (0.8) | 3 145 (0.7) | 2 685 (0.6) | 1 614 (0.6) | 9 611 (0.7) |
| | | | ***Baseline morbidity*** | | |
| *n with CVD (%)* | 8 025 (2.8) | 10 836 (2.5) | 13 836 (2.8) | 4 732 (1.4) | 37 429 (2.4) |
| *n with diabetes (%)* | 102 (0.04) | 255 (0.06) | 321 (0.06) | 273 (0.08) | 951 (0.06) |
| *n with kidney disease (%)* | 285 (0.1) | 399 (0.09) | 508 (0.1) | 316 (0.09) | 1 508 (0.1) |
| *n with malignant cancer (%)* | 71 (0.03) | 134 (0.03) | 371 (0.07) | 231 (0.07) | 807 (0.05) |
| *n with respiratory disease (%)* | 22 760 (8.04) | 49 808 (11.3) | 88 904 (17.9) | 66 155 (19.6) | 227 627 (14.6) |
| | | | ***Parental education*** | | |
| *Low n (%)* | 101 918 (52.9) | 141 205 (34.8) | 85 586 (17.5) | 28 186 (8.4) | 356 895 (25) |
| *Medium n (%)* | 77 020 (40) | 213 050 (52.5) | 309 246 (63.1) | 222 095 (65.8) | 821 411 (57.6) |
| *High n (%)* | 13 787 (7.2) | 51 660 (12.7) | 95 589 (19.5) | 87 069 (25.8) | 248 105 (17.4) |
| | | | ***COVID-19 diagnoses*** | | |
| *n in hospital due to COVID-19 (%)* | 728 (0.26) | 944 (0.21) | 674 (0.14) | 248 (0.07) | 2 594 (0.17) |
| *n in intensive care due to COVID-19 (%)* | 195 (0.07) | 209 (0.05) | 130 (0.03) | 25 (0.01) | 559 (0.04) |
| *n dead due to COVID-19 (%)* | 117 (0.04) | 43 (0.01) | 24 (0.00) | 0 | 184 (0.01) |

*Cardiorespiratory fitness in early adulthood and severity of COVID-19*

Our analysis shows a protective association between higher cardiorespiratory fitness at conscription and hospitalization, intensive care or death due to COVID-19 later in life, adjusted for examination year and place and conscript's age at examination. Odds ratios for high versus low fitness were 0.76 (95% CI 0.67-0.85), 0.61 (0.48-0.78) and 0.56 (0.37-0.85) respectively.

This association remains statistically significant with only minor attenuation after also controlling for BMI (figure 2, left panel), as well as blood pressure, height, baseline morbidity and parental education level (full results in table S2, supplementary materials).

[figure 2 about here]



For peer review only - http://bmjopen.bmj.com/site/about/guidelines.xhtml

In order to examine whether there was any additional or decreased protection at the highest level of CRF, categories 8 and 9 were disaggregated but no statistically significant difference could be seen between the two subgroups. Adjusting for muscle strength did not change the results for CRF.

Interaction analyses indicated that the associations between CRF and all three COVID-19 endpoints were not modified by weight status, blood pressure, baseline morbidity or education. For example, comparing high vs. low fitness in overweight (BMI group 3-5) and non-overweight conscripts (BMI group 1-2), the association with intensive care requiring COVID-19 tended to be stronger in the overweight group, OR = 0.49 (0.25-0.96), than in the non-overweight group, OR = 0.65 (0.50-0.85), but not significantly so (p-value for interaction = 0.5). Similarly the other three tested interactions were not statistically significant (p>0.1).

*Muscle strength and severity of COVID-19*

Muscular strength, a continuous variable measured in units of Newton and recorded until 1997, showed associations with COVID-19 that were similar in direction to the more robust protective association with cardiorespiratory fitness. As shown in figure 2, right panel, when analyzed in intervals of 313 N (=1 SD) the association was statistically significant for all three outcomes, but after controlling for cardiovascular fitness the results were attenuated (full results in table S3). Further analysis using cubic spline analyses confirmed the association between muscle strength and Covid-19. Specifically, these analyses, illustrated in supplementary materials (figure S1), confirmed that lower muscle strength in young adulthood has a significant linear association for all 3 endpoints, and no significant non-linear component.

*Supplementary mediation analysis*

A supplementary analysis of CVD as a mediating factor in the association between CRF or muscle strength and severe COVID was conducted. This analysis indicated that the direct effects of CRF and muscular strength (not mediated by CVD) are only slightly weaker than the overall total effect. Full results of this analysis are available in supplementary table S4.

**Discussion**

The main finding of this study is that lower cardiorespiratory fitness and lower muscle strength in young adulthood is predictive of severe illness and death due to COVID-19 15 to 52 years later. The protective association between cardiovascular fitness and COVID-19 severe enough to warrant hospitalization, intensive care and/or death was not attenuated by controlling for BMI at the time of conscription examinations. It was also independent of parental education, chronic morbidity and blood pressure at conscription.

As of this date no other comparable study has been published on the link between fitness in young adulthood and later COVID-19. Using data from the Swedish military conscript registry provided us with objective measures of fitness in a uniquely large sample. This together with the prospective design, the



For peer review only - http://bmjopen.bmj.com/site/about/guidelines.xhtml

For peer review only

long and near-complete follow-up from well validated hospital records and the death registry provides a strong ground for drawing conclusions.

Other studies point in the same direction, though with a shorter time span between exposure and outcome. For example measured hand grip strength have been showed to correlate with COVID-19 hospitalizations among adults 50 years and older(10). Also being consistently inactive is strongly associated with increased risk of severe COVID-19(7).

A possible mechanism for associations between CRF and severe COVID-19 could be higher cardiovascular morbidity (diagnosed or not) in the years after conscription examination among those with low cardiorespiratory fitness. Earlier studies using the same data have shown an association between low fitness and higher risk of heart failure, diabetes type 2, stroke, hypertension, ischemic heart disease and psychiatric disorders(17,25–28). In this regard, cardiorespiratory fitness could also be a marker for a better long-term lifestyle (less smoking, better diet).

Alternatively, there could be a direct anti-inflammatory effect or a modulation of the immune system, either acquired during adolescence and early adulthood and retained during the years leading up to 2020, or continually reinforced by physical activity established earlier in life (29). In a subsample of 139 609 individuals we had access to more recent CRF data collected from a health profiling registry(30), showing a clear correlation between these estimates of $VO2_{max}$ and the fitness categories from conscription, indicating that physical fitness tracks over time.

However, only a minor part of the protective association between CRF and COVID-19 outcome was mediated by protection from CVD in midlife. This points to a genuine effect of physical fitness acting via a modulation of the immune system. Of course, we cannot rule out a genetic predisposition to lower inflammatory levels, in conjunction with genetically higher cardiorespiratory fitness(31).

Despite a number of strengths, the study is not without limitations, including the fact that a large number of Swedish citizens have immigrated as adults and never went through the conscription examination. Immigrants have been overrepresented in the Swedish COVID-19 mortality and morbidity statistics with explanations ranging from crowded living conditions, lack of official information, and occupations where working from home is not an option. This group has a lower socioeconomic status and higher degree of lifestyle related risk factors (more obesity, more smoking, and more diabetes). Importantly, this group has also lower cardiorespiratory fitness, potentially contributing to explaining their higher risk(32).

Moreover, the results from this cohort can only be generalized to men, since the conscription process was not mandatory for women. Other limitations include some protocol changes in strength and CRF measurements over the years. For instance, a rather large number of conscripts are lacking CRF scores, predominantly during the last decade where the lowest scores were not recorded. This makes it difficult to draw conclusions regarding very low CRF levels and could underestimate the adverse association. In contrast to the CRF scores the muscular strength measurements among the conscripts are normally



For peer review only - http://bmjopen.bmj.com/site/about/guidelines.xhtml

distributed, and the analysis shows a considerable increase in the odds of developing severe COVID-19 among those at the lowest end of the distribution. When cardiorespiratory fitness was included in the model the association was weakened somewhat, indicating that CRF is the more important factor. Height turned out to be independently predictive of hospitalization with OR 1.014 (1.006-1.021) per cm (95% CI, basic model adjusted for BMI), with a possible explanation being the association between height and venous thromboembolism(33).

A related concern is the current age-range of the cohort (32-70 years in 2020). All reported findings were adjusted for exact age at conscription and year of conscription examination, and thus indirectly for age in 2020. To further illustrate this point, we repeated the analysis after stratification at median current age (52 years) and found only minor differences between the age groups. This underscores that protective associations of higher fitness are observed independent of cohort differences in current age or length of follow-up.

The present study has clear clinical implications. Physical fitness is a (largely) modifiable exposure and is therefore an important preventive measure both affecting later cardiovascular disease - and as this study shows - severe COVID-19. The obvious public health implications ought to be to further strengthen efforts concerning fitness in the young and presumably through the life course. Anecdotal stories about severely sick athletes during 2020 (the first patient in intensive care in Italy was a marathon runner) raised the question if extremely physically fit individuals had a higher risk of severe COVID-19 but no statistically significant difference could be seen between those of very high cardiorespiratory fitness (score 9) and those with moderately-high (score 8), although the power of this analysis was low.

Looking towards the future there are also the indirect effects of the pandemic and subsequent lock-downs on physical activity behaviors. In Sweden, a country with relatively few restrictions during the pandemic, only small changes in lifestyle habits have been shown so far(34), even though the amount of time sitting down is similar to other settings(35,36).

Considering the fact that women seem to run lower risk for severe COVID-19(**37**) it is unfortunate not to be able to compare the protective effects of cardiorespiratory fitness in women and men. Using other sources of data such as the Swedish medical birth register, this might be done in a future study. Finally, although we found the results for cardiorespiratory fitness were generally robust and unaffected by adjusting for covariates such as BMI, we cannot exclude the possibility of residual confounding by unmeasured factors. This includes later exposures and comorbidities occurring between conscription and 2020.

**Conclusion**

There is growing evidence of the importance of cardiorespiratory fitness on morbidity later in life(**30**), whereas this study shows fitness at a young age may influence the severity level of COVID-19 many years



For peer review only - http://bmjopen.bmj.com/site/about/guidelines.xhtml

later. In the near future, updates of the COVID-19 data will make it possible to understand these effects in more detail, but are unlikely to reverse the results.

The findings of the present study reinforce the need to promote regular physical activity early in life to increase the general cardiorespiratory fitness and muscular strength of the population, not only to decrease the risk of future cardiovascular events and other conditions but also to offer protection against potential consequences of coming viral pandemics.

**Captions for figures and tables**

**Figure 1**: Construction of analytic sample

**Table 1**: Description of analytical sample, by conscription decade.

**Figure 2**: Association between categories of cardiorespiratory fitness (CRF) and severe COVID-19 (left panel, n=1 143 670, hospitalizations=2 006, intensive care=445 and deaths=149), and between muscle strength as a continuous predictor and severe COVID-19 (right panel, OR per SD of muscle strength, n=1 161 827, hospitalizations=2 252, intensive care=514 and deaths=180). All regression models were made with Firth's bias correction and adjusted for year and place, as well as age and BMI at conscription examination.

[All numbers for this figure can be found in tables S1 and S2]

**Data availability statement**: The data used in this study is available on request from the Swedish National Board of Health and Welfare, the Swedish intensive care registry and the Swedish tax agency.

**Copyright statement**

"I, the Submitting Author has the right to grant and does grant on behalf of all authors of the Work (as defined in the author licence), an exclusive licence and/or a non-exclusive licence for contributions from authors who are: i) UK Crown employees; ii) where BMJ has agreed a CC-BY licence shall apply, and/or iii) in accordance with the terms applicable for US Federal Government officers or employees acting as part of their official duties; on a worldwide, perpetual, irrevocable, royalty-free basis to BMJ Publishing Group Ltd ("BMJ") its licensees.

The Submitting Author accepts and understands that any supply made under these terms is made by BMJ to the Submitting Author unless you are acting as an employee on behalf of your employer or a postgraduate student of an affiliated institution which is paying any applicable article publishing charge ("APC") for Open Access articles. Where the Submitting Author wishes to make the Work available on an Open Access basis (and intends to pay the relevant APC), the terms of reuse of such Open Access

For peer review only

shall be governed by a Creative Commons licence – details of these licences and which licence will apply to this Work are set out in our licence referred to above."

"the Corresponding Author has the right to grant on behalf of all authors and does grant on behalf of all authors, an exclusive licence (or non-exclusive for government employees) on a worldwide basis to the BMJ Publishing Group Ltd to permit this article (if accepted) to be published in BMJ editions and any other BMJPGL products and sublicenses such use and exploit all subsidiary rights, as set out in our licence."

**References**

1. Clark A, Jit M, Warren-Gash C, Guthrie B, Wang HH, Mercer SW, et al. Global, regional, and national estimates of the population at increased risk of severe COVID-19 due to underlying health conditions in 2020: a modelling study. Lancet Glob Health. 2020;8(8):e1003–17.

2. Hamer M, Gale CR, Kivimäki M, Batty GD. Overweight, obesity, and risk of hospitalization for COVID-19: A community-based cohort study of adults in the United Kingdom. Proc Natl Acad Sci. 2020;117(35):21011–3.

3. O'Driscoll M, Dos Santos GR, Wang L, Cummings DA, Azman AS, Paireau J, et al. Age-specific mortality and immunity patterns of SARS-CoV-2. Nature. 2021;590(7844):140–5.

4. Ross R, Blair S, Arena R, Church T, Després J, Franklin B, et al. American Heart Association Physical Activity Committee of the Council on Lifestyle and Cardiometabolic Health; Council on Clinical Cardiology; Council on Epidemiology and Prevention; Council on Cardiovascular and Stroke Nursing; Council on Functional Genomics and Translational Biology; Stroke Council. Importance of assessing cardiorespiratory fitness in clinical practice: a case for fitness as a clinical vital sign: a scientific statement from the American Heart Association. Circulation. 2016;134(24):e653–99.

5. Zbinden-Foncea H, Francaux M, Deldicque L, Hawley JA. Does high cardiorespiratory fitness confer some protection against pro-inflammatory responses after infection by SARS-CoV-2? Obesity. 2020;

6. Yates T, Razieh C, Zaccardi F, Rowlands AV, Seidu S, Davies MJ, et al. Obesity, walking pace and risk of severe COVID-19 and mortality: analysis of UK Biobank. Int J Obes. 2021;1–5.

7. Sallis R, Young DR, Tartof SY, Sallis JF, Sall J, Li Q, et al. Physical inactivity is associated with a higher risk for severe COVID-19 outcomes: a study in 48 440 adult patients. Br J Sports Med. 2021;

8. Salgado-Aranda R, Pérez-Castellano N, Núñez-Gil I, Orozco AJ, Torres-Esquivel N, Flores-Soler J, et al. Influence of Baseline Physical Activity as a Modifying Factor on COVID-19 Mortality: A Single-Center, Retrospective Study. Infect Dis Ther. 2021;1–14.



For peer review only - http://bmjopen.bmj.com/site/about/guidelines.xhtml

9. Brawner C, Ehrman J, Bole S, Kerrigan D, Parikh S, Lewis B, et al. Maximal exercise capacity is inversely related to hospitalization secondary to coronavirus disease 2019. In 2020.

10. Cheval B, Sieber S, Maltagliati S, Millet GP, Formánek T, Chalabaev A, et al. Muscle strength is associated with COVID-19 hospitalization in adults 50 years of age and older. MedRxiv. 2021;

11. Åberg MA, Pedersen NL, Torén K, Svartengren M, Bäckstrand B, Johnsson T, et al. Cardiovascular fitness is associated with cognition in young adulthood. Proc Natl Acad Sci. 2009;106(49):20906–11.

12. Gao M, Piernas C, Astbury NM, Hippisley-Cox J, O'Rahilly S, Aveyard P, et al. Associations between body-mass index and COVID-19 severity in 6· 9 million people in England: a prospective, community-based, cohort study. Lancet Diabetes Endocrinol. 2021;

13. Prince SA, Adamo KB, Hamel ME, Hardt J, Gorber SC, Tremblay M. A comparison of direct versus self-report measures for assessing physical activity in adults: a systematic review. Int J Behav Nutr Phys Act. 2008;5(1):1–24.

14. Berg C, Rosengren A, Aires N, Lappas G, Toren K, Thelle D, et al. Trends in overweight and obesity from 1985 to 2002 in Göteborg, West Sweden. Int J Obes. 2005;29(8):916–24.

15. Nordesjö L, Schéle R. Validity of an ergometer cycle test and measures of isometric muscle strength when prediction some aspects of military performance. Swed J Def Med. 1974;10:11–23.

16. Lissner L, Mehlig K, Rosengren A, Torén K, Åberg MAI. A Growing Social Divide in Body Mass Index, Strength, and Fitness of Swedish Male Conscripts. J Adolesc Health 2019 Vol 65 Iss 2 Pp 232-238. 2019;65(2):232–8.

17. Åberg ND, Kuhn HG, Nyberg J, Waern M, Friberg P, Svensson J, et al. Influence of cardiovascular fitness and muscle strength in early adulthood on long-term risk of stroke in Swedish men. Stroke. 2015;46(7):1769–76.

18. World Health Organization. The Asia-Pacific perspective: redefining obesity and its treatment. 2000;

19. Cole TJ, Lobstein T. Extended international (IOTF) body mass index cut-offs for thinness, overweight and obesity. Pediatr Obes. 2012;7(4):284–94.

20. Williams B, Mancia G, Spiering W, Agabiti Rosei E, Azizi M, Burnier M, et al. 2018 ESC/ESH Guidelines for the management of arterial hypertension: The Task Force for the management of arterial hypertension of the European Society of Cardiology (ESC) and the European Society of Hypertension (ESH). Eur Heart J. 2018;39(33):3021–104.

21. Ludvigsson JF, Andersson E, Ekbom A, Feychting M, Kim J-L, Reuterwall C, et al. External review and validation of the Swedish national inpatient register. BMC Public Health. 2011;11(1):1–16.



For peer review only - http://bmjopen.bmj.com/site/about/guidelines.xhtml

22. King G, Zeng L. Logistic regression in rare events data. Polit Anal. 2001;9(2):137–63.

23. Li R, Hertzmark E, Louie M, Chen L, Spiegelman D. The sas lgtphcurv9 macro. Boston Channing Lab. 2011;

24. Valeri L, VanderWeele TJ. Mediation analysis allowing for exposure–mediator interactions and causal interpretation: theoretical assumptions and implementation with SAS and SPSS macros. Psychol Methods. 2013;18(2):137.

25. Lindgren M, Åberg M, Schaufelberger M, Åberg D, Schiöler L, Torén K, et al. Cardiorespiratory fitness and muscle strength in late adolescence and long-term risk of early heart failure in Swedish men. Eur J Prev Cardiol. 2017;24(8):876–84.

26. Crump C, Sundquist J, Winkleby MA, Sundquist K. Interactive effects of obesity and physical fitness on risk of ischemic heart disease. Int J Obes. 2017;41(2):255–61.

27. Crump C, Sundquist J, Winkleby MA, Sundquist K. Interactive effects of physical fitness and body mass index on the risk of hypertension. JAMA Intern Med. 2016;176(2):210–6.

28. Crump C, Sundquist J, Winkleby MA, Sieh W, Sundquist K. Physical fitness among Swedish military conscripts and long-term risk for type 2 diabetes mellitus: a cohort study. Ann Intern Med. 2016;164(9):577–84.

29. Gleeson M, Bishop NC, Stensel DJ, Lindley MR, Mastana SS, Nimmo MA. The anti-inflammatory effects of exercise: mechanisms and implications for the prevention and treatment of disease. Nat Rev Immunol. 2011;11(9):607–15.

30. Ekblom-Bak E, Ekblom B, Söderling J, Börjesson M, Blom V, Kallings LV, et al. Sex- and age-specific associations between cardiorespiratory fitness, CVD morbidity and all-cause mortality in 266.109 adults. Prev Med. 2019;127:105799.

31. Burtscher J, Millet GP, Burtscher M. Low cardiorespiratory and mitochondrial fitness as risk factors in viral infections: implications for COVID-19. 2020;

32. Lindgren M, Börjesson M, Ekblom Ö, Bergström G, Lappas G, Rosengren A. Physical activity pattern, cardiorespiratory fitness, and socioeconomic status in the SCAPIS pilot trial—a cross-sectional study. Prev Med Rep. 2016;4:44–9.

33. Brækkan SK, Borch KH, Mathiesen EB, Njølstad I, Wilsgaard T, Hansen J-B. Body height and risk of venous thromboembolism: the Tromsø Study. Am J Epidemiol. 2010;171(10):1109–15.

34. Blom V, Lönn A, Ekblom B, Kallings LV, Väisänen D, Hemmingsson E, et al. Lifestyle habits and mental health in light of the two COVID-19 pandemic waves in Sweden, 2020. Int J Environ Res Public Health. 2021;18(6):3313.

35. Cheval B, Sivaramakrishnan H, Maltagliati S, Fessler L, Forestier C, Sarrazin P, et al. Relationships between changes in self-reported physical activity, sedentary behaviour and health during the coronavirus (COVID-19) pandemic in France and Switzerland. J Sports Sci. 2020;1–6.



For peer review only - http://bmjopen.bmj.com/site/about/guidelines.xhtml

36. Flanagan EW, Beyl RA, Fearnbach SN, Altazan AD, Martin CK, Redman LM. The impact of COVID-19 stay-at-home orders on health behaviors in adults. Obesity. 2021;29(2):438–45.

37. Peckham H, de Gruijter NM, Raine C, Radziszewska A, Ciurtin C, Wedderburn LR, et al. Male sex identified by global COVID-19 meta-analysis as a risk factor for death and ITU admission. Nat Commun. 2020;11(1):1–10.

For peer review only



For peer review only - http://bmjopen.bmj.com/site/about/guidelines.xhtml

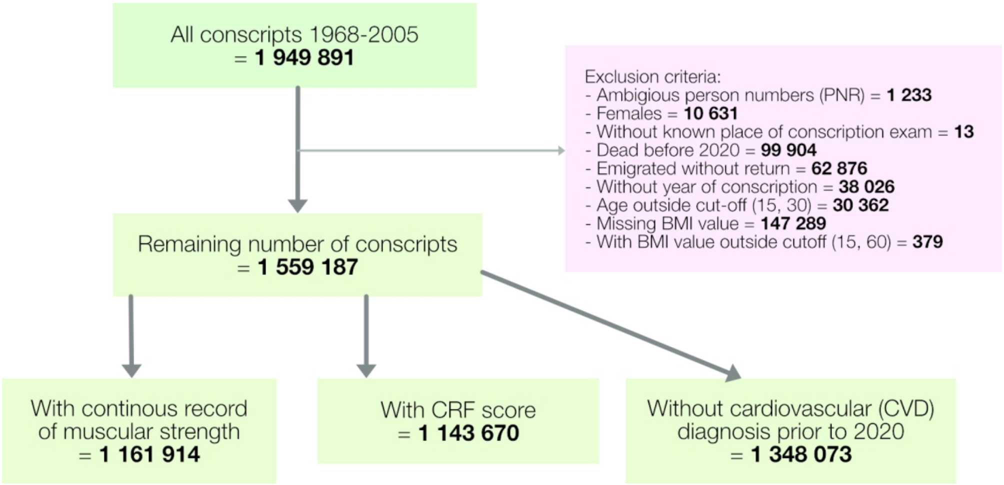


Figure 1: Construction of analytic sample

154x73mm (300 x 300 DPI)

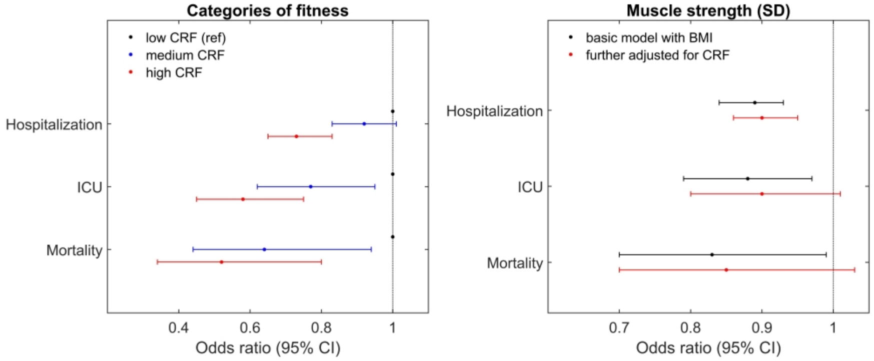


Association between categories of cardiorespiratory fitness (CRF) and severe COVID-19 (left panel, n=1 143 670, hospitalizations=2 006, intensive care=445 and deaths=149), and between muscle strength as a continuous predictor and severe COVID-19 (right panel, OR per SD of muscle strength, n=1 161 827, hospitalizations=2 252, intensive care=514 and deaths=180). All regression models were made with Firth's bias correction and adjusted for year and place, as well as age and BMI at conscription examination.

3333x1219mm (72 x 72 DPI)

## Supplementary material

**Table S1.** Included main diagnoses divided by categories when COVID-19 is secondary diagnosis.

| Included categories | ICD-codes |
|---|---|
| **COVID-related symptoms** | |
| Cough | R05 |
| Abnormalities of breathing | R06 |
| Pain in throat and chest | R07 |
| Other symptoms and signs involving the circulatory and respiratory system | R09 |
| Dizziness and giddiness | R42 |
| Fever of other or unknown origin | R50 |
| Headache | R51 |
| Malaise and fatigue | R53 |
| Syncope and collapse | R55 |
| **Upper and lower respiratory tract infections** | |
| Acute nasopharyngitis | J00 |
| Acute tonsillitis | J03 |
| Acute upper respiratory infections of multiple and unspecified sites | J06 |
| Influenza due to other identified influenza virus | J10 |
| Other viral pneumonia | J128 |
| Viral pneumonia, unspecified | J129 |
| Pneumonia due to Streptococcus pneumoniae | J13 |
| Bacterial pneumonia, not elsewhere classified | J15 |
| Pneumonia due to other specified infectious organisms | J168 |
| Pneumonia in diseases classified elsewhere | J17 |
| Pneumonia, unspecified organism | J18 |
| Unspecified acute lower respiratory infection | J22 |
| Coronavirus infection, unspecified | B342 |
| Other viral infections of unspecified site | B348 |
| Viral infection, unspecified | B349 |
| Coronavirus as the cause of diseases classified elsewhere | B972 |
| Other and unspecified infectious diseases | B99 |
| **Respiratory disorders** | |
| Pulmonary embolism | I26 |
| Acute respiratory distress syndrome | J80 |
| Pulmonary edema | J81 |
| Pleural effusion not elsewhere classified | J90 |
| Respiratory failure, not elsewhere classified | J96 |
| Respiratory disorders in diseases classified elsewhere | J99 |
| **Obstructive Airway Diseases** | |
| Acute bronchitis due to other specified organisms | J208 |
| Acute bronchitis, unspecified | J209 |
| Acute bronchiolitis due to other specified organisms | J218 |
| Acute bronchiolitis, unspecified | J219 |
| Other chronic obstructive pulmonary disease | J44 |
| Asthma | J45 |
| **Cardiac diseases** | |
| Viral carditis | B332 |
| Chronic ischemic heart disease | I25 |
| Acute pericarditis | I30 |
| Pericarditis in diseases classified elsewhere | I32 |
| Acute myocarditis, unspecified | I40 |
| Myocarditis in diseases classified elsewhere | I41 |
| Atrial fibrillation and flutter | I48 |

For peer review only

| | |
|---|---|
| Heart failure | I50 |
| Abnormalities of heart beat | R00 |
| **Kidney disorders** | |
| Acute kidney failure | N17 |
| Chronic kidney disease | N18 |
| Unspecified kidney failure | N19 |
| **Electrolyte disorders** | |
| Other disorders of fluid, electrolyte and acid-base balance | E87 |

For peer review only

**Table S2:** Results of main analysis. Association between conscript's fitness at time of examination and hospitalization, intensive care and death due to COVID-19 (OR, 95% CI, Firth's bias correction).

| *Fitness level* | **Basic model[a] n=1 143 670** | **Basic model controlled for BMI[b] n=1 143 670** | **Basic model controlled for blood pressure n=1 140 514** | **Basic model controlled for baseline morbidity[c] n=1 143 670** | **Basic model controlled for BMI[2] and height N=1 143 670** | **Basic model controlled for parental education n=1 031 246** | **Basic model controlled for BMI, height, blood pressure, morbidity and parental education n=1 028 122** |
|---|---|---|---|---|---|---|---|
| | **All hospitalization due to COVID-19.** | | | | | | |
| | cases=2 006 | cases=2 006 | cases=2 003 | cases=2 006 | cases=2 006 | cases=1 710 | cases=1 707 |
| *Low (1-5)* | **1** | **1** | **1** | **1** | **1** | **1** | **1** |
| *Medium (6-7)* | 0.91 (0.82-1.01) | 0.92 (0.83-1.01) | 0.91 (0.82-1.01) | 0.91 (0.82-1.01) | 0.91 (0.82-1.01) | 0.91 (0.82-1.02) | 0.91 (0.81-1.02) |
| *High (8-9)* | **0.76** (0.67-0.85) | **0.73** (0.65-0.83) | **0.76** (0.68-0.85) | **0.76** (0.67-0.85) | **0.71** (0.63-0.81) | **0.74** (0.65-0.85) | **0.73** (0.64-0.83) |
| *AUROC* | 0.66 | 0.66 | 0.66 | 0.66 | 0.66 | 0.67 | 0.67 |
| | **Intensive care due to COVID-19** | | | | | | |
| | cases=445 | cases=445 | cases=445 | cases=445 | cases=445 | cases=363 | cases=363 |
| *Low (1-5)* | **1** | **1** | **1** | **1** | **1** | **1** | **1** |
| *Medium (6-7)* | **0.76** (0.62-0.95) | **0.77** (0.62-0.95) | **0.76** (0.62-0.95) | **0.77** (0.62-0.95) | **0.76** (0.61-0.94) | **0.76** (0.60-0.96) | **0.76** (0.60-0.96) |
| *High (8-9)* | **0.61** (0.48-0.78) | **0.58** (0.45-0.75) | **0.61** (0.48-0.78) | **0.62** (0.48-0.79) | **0.57** (0.44-0.74) | **0.60** (0.45-0.80) | **0.60** (0.46-0.80) |
| *AUROC* | 0.69 | 0.71 | 0.70 | 0.69 | 0.71 | 0.71 | 0.71 |
| | **Mortality due to COVID-19** | | | | | | |
| | cases=149 | cases=149 | cases=149 | cases=149 | cases=149 | cases= 111 | cases=111 |
| *Low (1-5)* | **1** | **1** | **1** | **1** | **1** | **1** | **1** |
| *Medium (6-7)* | **0.67** (0.46-0.96) | **0.64** (0.44-0.94) | **0.67** (0.47-0.97) | **0.66** (0.46-0.96) | **0.63** (0.43-0.92) | **0.53** (0.34-0.83) | **0.53** (0.34-0.81) |
| *High (8-9)* | **0.56** (0.37-0.85) | **0.52** (0.34-0.80) | **0.57** (0.38-0.87) | **0.56** (0.37-0.85) | **0.50** (0.32-0.77) | **0.52** (0.32-0.83) | **0.50** (0.31-0.81) |
| *AUROC* | 0.81 | 0.82 | 0.81 | 0.81 | 0.82 | 0.82 | 0.82 |

[a] adjusted for year and place of examination, and age at examination.
[b] BMI, $BMI^2$, $BMI^3$
[c] including CVD, diabetes, kidney disease, respiratory disease and cancer.

For peer review only

**Table S3:** Association between muscle strength at conscription and severity of COVID disease (OR for disease by 1 SD higher muscle strength (SD = 313 N), 95% CI, Firth's bias correction)

| Outcome | Total n | Cases | OR (95% CI) | AUROC |
|---|---|---|---|---|
| *Hospital*[a] | 1 161 914 | 2 252 | **0.89** (0.84-0.93) | 0.64 |
| *ICU*[a] | | 514 | **0.88** (0.79-0.97) | 0.68 |
| *Mortality*[a] | | 180 | **0.83** (0.70-0.99) | 0.80 |
| | | | | |
| *Hospital*[b] | 952 225 | 1 850 | **0.90** (0.86-0.95) | 0.64 |
| *ICU*[b] | | 421 | 0.90 (0.80-1.01) | 0.68 |
| *Mortality*[b] | | 146 | 0.85 (0.70-1.03) | 0.79 |

[a] basic model adjusted for BMI, $BMI^2$, $BMI^3$ and height.
[b] above model further adjusted for fitness categories.

For peer review only

**Figure S1**: Results of restricted cubic spline analyses of continuous muscle strength and severity of COVID-19 adjusted for age, year, and place of conscript examination, BMI, BMI$^2$, BMI$^3$, and height (total number of observations = 1 161 914).

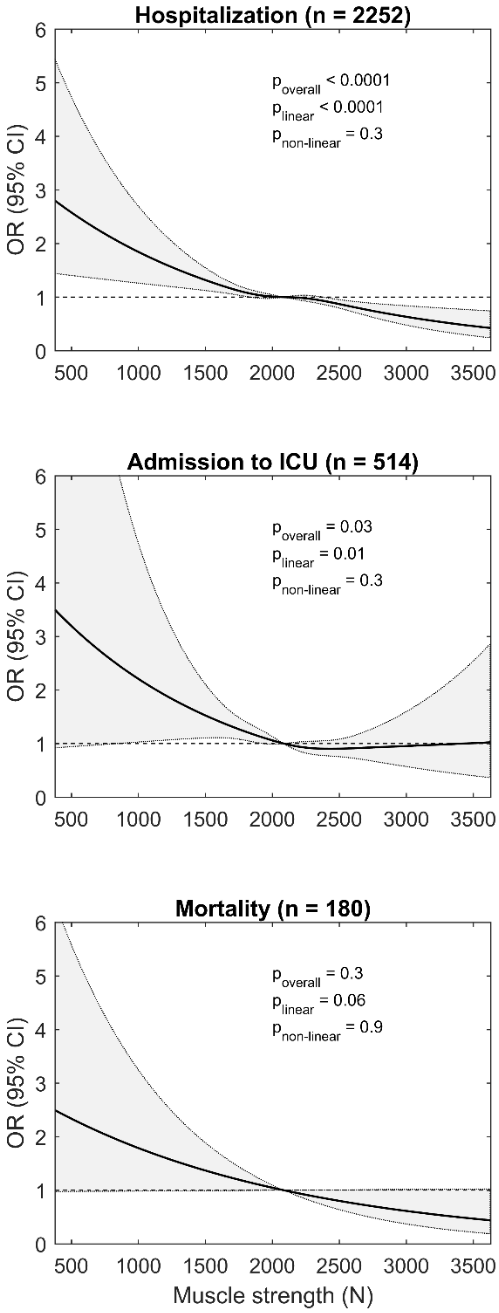


review only

**Table S4.** Results of mediation analysis between cardiovascular fitness, muscle strength, cardiovascular disease and severe COVID-19 (OR with 95%CI).

| Outcome | Direct | Indirect | Total | Proportion-mediated[c] |
|---|---|---|---|---|
| | **Muscle strength[a]** | | | |
| *Hospital* | **0.893** (0.850-0.937) | **0.995** (0.994-0.996) | **0.888** (0.846-0.933) | 4.0 % |
| *ICU* | **0.880** (0.796-0.973) | **0.997** (0.995-0.998) | **0.877** (0.793-0.970) | 2.2 % |
| *Mortality* | 0.855 (0.720-1.015) | **0.992** (0.989-0.995) | 0.848 (0.714-1.007) | 4.5 % |
| | **CRF[a,b]** | | | |
| *Hospital[a]* | **0.860** (0.810-0.913) | **0.987** (0.985-0.989) | **0.849** (0.800-0.901) | 7.4 % |
| *ICU[a]* | **0.772** (0.679-0.877) | **0.991** (0.987-0.995) | **0.765** (0.673-0.869) | 3.0 % |
| *Mortality[a]* | **0.725** (0.578-0.909) | **0.978** (0.969-0.986) | **0.709** (0.566-0.889) | 5.5 % |

[a] adjusted for age, year, and place of conscript examination, BMI, $BMI^2$, $BMI^3$ and height.
[b] treating the 3-level variable for CRF as ordinal score.
[c] calculated from direct and indirect effect odds ratios as $OR^{direct}(OR^{indirect}-1)/(OR^{direct}OR^{indirect}-1)$.

VanderWeele T, Mediation Analysis: A Practioner's Guide, Annu. Rev. Public Health 2016.37:17-32

For peer review only

STROBE Statement—Checklist of items that should be included in reports of ***cohort studies***

| | Item No | Recommendation | Page No |
|---|---|---|---|
| **Title and abstract** | 1 | (*a*) Indicate the study's design with a commonly used term in the title or the abstract<br>(*b*) Provide in the abstract an informative and balanced summary of what was done and what was found | 4 |
| **Introduction** | | | |
| Background/rationale | 2 | Explain the scientific background and rationale for the investigation being reported | 5 |
| Objectives | 3 | State specific objectives, including any prespecified hypotheses | 5 |
| **Methods** | | | |
| Study design | 4 | Present key elements of study design early in the paper | 5 |
| Setting | 5 | Describe the setting, locations, and relevant dates, including periods of recruitment, exposure, follow-up, and data collection | 6 |
| Participants | 6 | (*a*) Give the eligibility criteria, and the sources and methods of selection of participants. Describe methods of follow-up<br>(*b*) For matched studies, give matching criteria and number of exposed and unexposed | 7 |
| Variables | 7 | Clearly define all outcomes, exposures, predictors, potential confounders, and effect modifiers. Give diagnostic criteria, if applicable | 7-8 |
| Data sources/ measurement | 8* | For each variable of interest, give sources of data and details of methods of assessment (measurement). Describe comparability of assessment methods if there is more than one group | 5-6 |
| Bias | 9 | Describe any efforts to address potential sources of bias | 5 |
| Study size | 10 | Explain how the study size was arrived at | 7 |
| Quantitative variables | 11 | Explain how quantitative variables were handled in the analyses. If applicable, describe which groupings were chosen and why | 6-7 |
| Statistical methods | 12 | (*a*) Describe all statistical methods, including those used to control for confounding<br>(*b*) Describe any methods used to examine subgroups and interactions<br>(*c*) Explain how missing data were addressed<br>(*d*) If applicable, explain how loss to follow-up was addressed<br>(*e*) Describe any sensitivity analyses | 8 |
| **Results** | | | |
| Participants | 13* | (a) Report numbers of individuals at each stage of study—eg numbers potentially eligible, examined for eligibility, confirmed eligible, included in the study, completing follow-up, and analysed<br>(b) Give reasons for non-participation at each stage<br>(c) Consider use of a flow diagram | 7, 9 |
| Descriptive data | 14* | (a) Give characteristics of study participants (eg demographic, clinical, social) and information on exposures and potential confounders<br>(b) Indicate number of participants with missing data for each variable of interest<br>(c) Summarise follow-up time (eg, average and total amount) | 9 |
| Outcome data | 15* | Report numbers of outcome events or summary measures over time | 9 |

For peer review only


For peer review only - http://bmjopen.bmj.com/site/about/guidelines.xhtml

| | | | |
|---|---|---|---|
| Main results | 16 | (*a*) Give unadjusted estimates and, if applicable, confounder-adjusted estimates and their precision (eg, 95% confidence interval). Make clear which confounders were adjusted for and why they were included<br>(*b*) Report category boundaries when continuous variables were categorized<br>(*c*) If relevant, consider translating estimates of relative risk into absolute risk for a meaningful time period | 9 |
| Other analyses | 17 | Report other analyses done—eg analyses of subgroups and interactions, and sensitivity analyses | 9-10 |
| **Discussion** | | | |
| Key results | 18 | Summarise key results with reference to study objectives | 10 |
| Limitations | 19 | Discuss limitations of the study, taking into account sources of potential bias or imprecision. Discuss both direction and magnitude of any potential bias | 10 |
| Interpretation | 20 | Give a cautious overall interpretation of results considering objectives, limitations, multiplicity of analyses, results from similar studies, and other relevant evidence | 10 |
| Generalisability | 21 | Discuss the generalisability (external validity) of the study results | 10 |
| **Other information** | | | |
| Funding | 22 | Give the source of funding and the role of the funders for the present study and, if applicable, for the original study on which the present article is based | 4 |

*Give information separately for exposed and unexposed groups.

**Note:** An Explanation and Elaboration article discusses each checklist item and gives methodological background and published examples of transparent reporting. The STROBE checklist is best used in conjunction with this article (freely available on the Web sites of PLoS Medicine at http://www.plosmedicine.org/, Annals of Internal Medicine at http://www.annals.org/, and Epidemiology at http://www.epidem.com/). Information on the STROBE Initiative is available at http://www.strobe-statement.org.

For peer review only
